# A Low-Profile Self-Stealth Programmable Metasurface with In-Band and Out-of-Band RCS Reduction

**DOI:** 10.34133/research.1017

**Published:** 2025-12-04

**Authors:** Yajie Mu, Jiaqi Han, Lihao Zhu, Dexiao Xia, Xiangjin Ma, Haixia Liu, Long Li

**Affiliations:** Key Laboratory of High-Speed Circuit Design and EMC of Ministry of Education, School of Electronic Engineering, Xidian University, Xi’an 710071, China.

## Abstract

Programmable metasurfaces show great application potential in many fields, so studying their radar cross-section reduction (RCSR) characteristics is very important. Metasurfaces based on polarization conversion and artificial magnetic conductors are widely used for RCSR, but they cannot scan beams. Programmable metasurfaces can dynamically steer beams in-band and reduce RCS by changing the phase of electromagnetic waves. But to meet practical needs, they must be stealthy both in-band and out-of-band. This paper presents a low-profile self-stealth programmable metasurface. Self-stealth describes the capability of the metasurface to achieve in-band and out-of-band RCSR through its intrinsic design, without requiring additional structures such as radomes or absorbing materials. The design principle is to achieve out-of-band RCS reduction without increasing the profile height or adding extra radio frequency components. The meta-atom has a positive-intrinsic-negative (PIN) diode to control the in-band (*f*_0_) *x*-polarization phase. Switching the PIN diode states gives 1-bit phase modulation. The in-band *y*-polarization uses a bridge design. Changing the size can get 1-bit phase, and the *x*- and *y*-polarizations can be adjusted separately. Also, the top layer has 4 cross-shaped dumbbells and 4 branch structures for out-of-band high (*fh*_1_) and low (*fl*_1_) frequencies. The middle layer has four 45° rotated cross-shaped dumbbells and 4 branch structures for out-of-band high (*fh*_2_) and low (*fl*_2_) frequencies. So, the meta-atom can modulate phase across 5 frequency bands (*fl*_2_ < *fl*_1_ < *f*_0_ < *fh*_2_ < *fh*_1_). The profile height is 0.065λ of the in-band center frequency, which is more conducive to conformal design. A 12 × 12 prototype was made. Simulation and measurement show that the metasurface reduces RCS in 5 bands and its in-band radiation can be used in communication transmission mode, which is good for programmable metasurface technology development.

## Introduction

Radar cross-section reduction (RCSR) is crucial for stealth technology. By reducing RCS, the probability of a target being detected by radar can be greatly decreased. This, in turn, enhances the survivability of the equipment. In recent years, programmable metasurfaces, characterized by their low cost, low loss, programmability, and ease of deployment, have emerged as a promising candidate for 6G wireless communication systems and radar systems. Therefore, the study of in-band and out-of-band RCS characteristics of programmable metasurfaces is particularly important.

Over the past 2 decades, metasurfaces have garnered significant attention due to their multifunctionality. Cui et al. [1,2] pioneered the integration of switchable components on metasurfaces, giving rise to programmable metasurfaces. This innovation enables metasurfaces to dynamically interact with electromagnetic waves (EMs) in real time. Programmable metasurfaces offer remarkable capabilities through the dynamic control of various physical quantities, such as phase [3–5], polarization [6–9], frequency [10,11], amplitude [12–14], and position [15]. These manipulations allow programmable metasurfaces to achieve diverse functionalities, including anomalous reflection and refraction. To attain multidimensional control over EMs, programmable metasurfaces have incorporated multiple dynamic physical quantities, such as phase-amplitude [16–18], phase-time [19], phase-space [20–23], amplitude-time [24], and spatiotemporal frequency [25]. For instance, dynamic control of EMs across the entire space is achieved by utilizing spatial and phase modulation [21]. Additionally, controlling both phase and amplitude enables real-time adjustment of the amplitude and direction of EMs [24]. Furthermore, studies have successfully reduced RCS and achieved stealth effects by modulating time and phase [19]. However, existing programmable metasurfaces can dynamically steer beams and exhibit stealth characteristics within the in-band. In practical applications, it is crucial for programmable metasurfaces to demonstrate stealth performance both in-band and out-of-band. Achieving in-band and out-of-band stealth with programmable metasurfaces remains a challenging issue that warrants further research and exploration, making it a significant topic in current programmable metasurface research.

Recent years have seen extensive research into the radiation characteristics of programmable metasurfaces, with a focus on reflection [26–28], transmission [29,30], and reflection–transmission [31,32] types. For example, a 2-bit circularly polarized programmable reflectarray has demonstrated a ±60° scanning range and a sidelobe level under −20 dB [26]. Likewise, a dual-polarization 1-bit programmable transmitarray has shown ±50° beam-scanning capabilities [29]. However, research on the stealth capabilities of programmable reflection and transmission metasurface arrays remains limited, necessitating further exploration to enhance these functionalities. Various stealth-achieving methods have been explored, such as using absorbing materials or structures [33–35], modifying antenna structure [36], and employing scattering cancellation [37–43]. Scattering cancellation primarily utilizes metasurface structures like artificial magnetic conductors and polarization–conversion metasurfaces. While metasurfaces based on polarization conversion and artificial magnetic conductors are commonly used for RCS reduction, they lack beam-scanning capabilities.

Numerous studies have been conducted on the in-band stealth characteristics of programmable metasurfaces [44–49]. A 1-bit programmable metasurface array has been proposed [44]. It enables 2-dimensional beam scanning in *x*-polarization and achieves in-band RCS reduction via *y*-polarization scattering cancellation. A spatiotemporal metasurface for in-band scattering control has also been proposed [47]. It can reshape scattering patterns in both spatial and spectral domains. Additionally, a programmable metasurface with 2 switchable modes, reflective and tunable absorptive, has been designed [48]. The metasurface has 1-bit phase resolution. The positive-intrinsic-negative (PIN) diode’s variable resistance in its on-state allows the tunable absorption mode for in-band RCS reduction. However, there is relatively little research on the out-of-band stealth characteristics of programmable metasurfaces. The utilization of absorbing frequency selective reflection (AFSR) or absorbing frequency selective transmission (AFST) surfaces as ground planes or radomes to achieve out-of-band stealth for programmable metasurfaces presents some challenges, such as introducing losses, integration difficulties, and high profile. In the out-of-band, AFSR and AFST exhibit absorbing properties, achieving out-of-band stealth. The programmable metasurface comprises an active coding metasurface cascaded with an absorptive metasurface radome [50]. The out-of-band stealth is obtained owing to the absorptive metasurface’s absorption characteristics. The radome consists of 2 types of passive meta-atoms, which can also achieve in-band stealth [51]. However, to ensure that the absorbing radome does not affect the metasurface performance, the total profile height is approximately 0.51λ at the center frequency [50], equivalent to 42.5 mm. Further research is needed to explore the potential of programmable metasurfaces for achieving stealth beyond the operating band. In particular, there is a need for low-profile designs that can achieve out-of-band RCS reduction on programmable metasurfaces.

Here, we proposed a low-profile self-stealth programmable metasurface. Self-stealth refers to the capability of the metasurface to achieve in-band and out-of-band RCSR through its intrinsic design. The metasurface consists of meta-atoms, with each meta-atom containing a PIN diode that dynamically controls the phase of the in-band (*f*_0_) *x*-polarization. By switching the PIN diode states, the meta-atom achieves a 1-bit phase. The in-band *y*-polarization adopts a bridge design, which can achieve a 1-bit phase by changing the size of *y*-polarization, and the *x*-polarization and *y*-polarization can be adjusted independently. In addition, 4 cross-shaped dumbbells and 4 branch structures are introduced at the top layer of the meta-atom to control the out-of-band high (*fh*_1_) and low (*fl*_1_) frequencies, respectively. To further enhance the bandwidth of the 1-bit phase for the out-of-band, the 4 cross-shaped dumbbell structures (*fh*_2_) and branch structures (*fl*_2_) are added to the middle layer, where dumbbell structures are rotated by 45°. Therefore, the proposed meta-atom achieves phase modulation in 5 frequency bands (*fl*_2_ < *fl*_1_ < *f*_0_ < *fh*_2_ < *fh*_1_). Compared to existing in-band and out-of-band RCS reduction programmable metasurfaces with a profile of 0.51λ, our proposed metasurface has a much lower profile of only 0.065λ. To validate the functionality, a 12 × 12 prototype is designed and fabricated. Simulation and measured results confirm that the proposed metasurface possesses RCS reduction for 5 frequency bands and its radiation properties in the in-band can be utilized for communication transmission mode.

## Results and Discussion

**Fig. 1.**
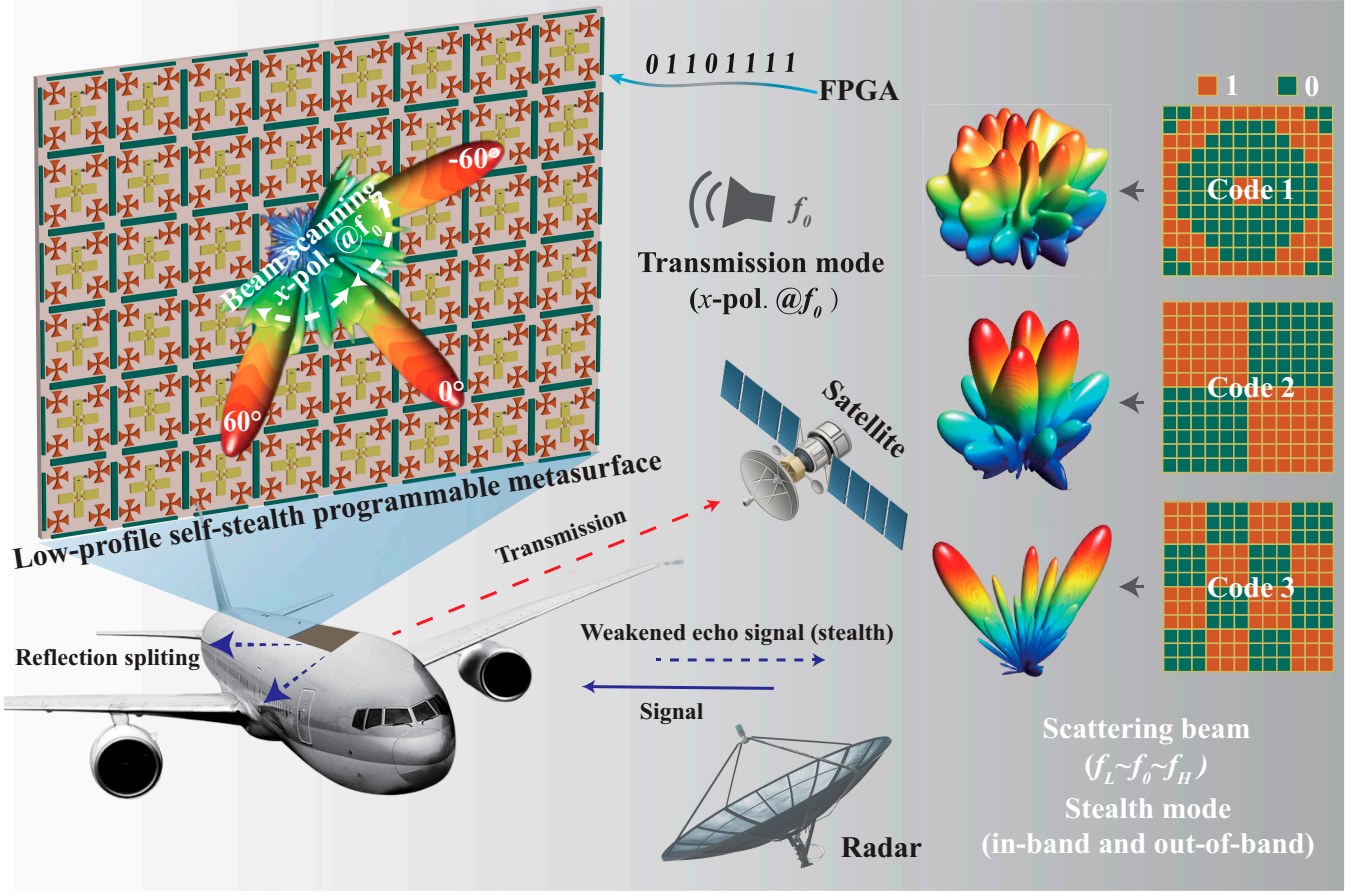
Conceptual illustration of the proposed low-profile self-stealth programmable metasurface. The metasurface exhibits radiation and stealth characteristics in the in-band co-polarization. It also demonstrates stealth characteristics in the in-band cross-polarization and out-of-band full-polarization.

Figure 1 illustrates the principle of the low-profile self-stealth programmable metasurface. Normally, aircraft communication equipment requires a radome with absorbing properties in out-of-band to achieve stealth and avoid detection by radar. However, this approach reduces its own signal strength and adds complexity to the system. In contrast, our proposed low-profile self-stealth programmable metasurface offers both in-band and out-of-band stealth capabilities without compromising communication quality. It ensures excellent transmission performance with ground or satellite stations while providing self-stealth features against radar tracking and detection. More importantly, it does not require a radome, achieving a low profile and thus allowing for easier conformal integration with an aircraft.

The programmable metasurface is composed of meta-atoms, each containing a PIN diode that controls the phase of the in-band (*f*_0_) *x*-polarization. By switching the PIN diode between ON and OFF states, the meta-atom achieves a 180° phase difference, allowing for 1-bit phase modulation. These meta-atoms are organized to form a 2-dimensional programmable metasurface, and their coding sequences are controlled by an field programmable gate array (FPGA). When excited by a horn antenna in the near-field region, the metasurface enables dynamic beam scanning within the in-band, facilitating communication transmission mode and other applications. Furthermore, the metasurface exhibits scattering cancellation properties for *x*-polarized incident plane waves, thus achieving stealth characteristics for the in-band co-polarization.

For the in-band *y*-polarization and out-of-band (*f*_L_ and *f*_H_) full polarization, we employ 2 different sizes of meta-atoms to achieve a 180° phase difference, enabling 1-bit phase modulation. These meta-atoms are arranged in a specific configuration, as depicted in codes 1, 2, and 3 in Fig. 1. When excited by a plane wave, the resulting scattering cancellation provides stealth functionality. By simultaneously controlling the state of the PIN diodes and adjusting the size of the meta-atoms, an in-band and out-of-band self-stealth programmable metasurface is ultimately realized. It eliminates the need for additional radomes or absorbing materials.

### Meta-atom design

**Fig. 2.**
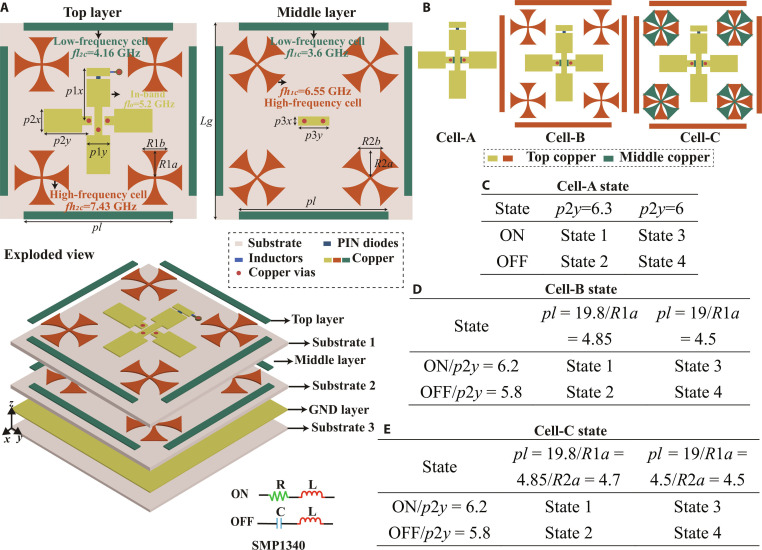
Meta-atom design. (A) Geometry of the meta-atom. (B) Design steps for the meta-atom. States of (C) Cell-A, (D) Cell-B, and (E) Cell-C.

The proposed programmable metasurface realizes self-stealth by scattering cancellation. To achieve this goal, the meta-atom needs to possess multiple 1-bit phase modulation bandwidths, both in-band and out-of-band. Additionally, independent control of *x*- and *y*-polarizations within the desired frequency range is necessary. To accomplish these objectives, we propose a novel meta-atom structure as depicted in Fig. 2A. The meta-atom consists of 4 metal layers and 3 dielectric substrates. The dielectric substrates are made of Shengyi S7136H with a relative permittivity of 3.55 and a loss tangent of 0.003. The thickness of substrates 1 and 2 is 1.524 mm, while substrate 3 has a thickness of 0.5 mm. The 4 metal layers are arranged in the following order from top to bottom: top layer, middle layer, ground layer, and direct current (DC) feedline layer. The top layer consists of 3 different components. Among them, the 4 branch structures belong to the first out-of-band low-frequency band (*fl*_1_), while the 4 cross-shaped dumbbell structures belong to the first out-of-band high-frequency band (*fh*_1_). Additionally, the cross structure corresponds to the in-band. A PIN diode is integrated with the *x*-polarization branch. The model of this PIN diode is SMP1340, and its equivalent circuit is shown in Fig. 2A. By controlling the ON and OFF states of the PIN diode, a phase difference of 180° can be achieved in the *x*-polarization, enabling 1-bit phase control. However, there is no PIN diode integrated on the *y*-polarization branch. Moreover, the branches in the *x*- and *y*-polarizations are not directly connected at the center position of the top layer. Instead, they are isolated from each other and their characteristics are unaffected by employing a bridge design, as shown in Fig. 2A. The middle layer also contains 4 metal branches and 4 cross-shaped dumbbell elements rotated by 45°. They correspond to the second out-of-band low-frequency band (*fl*_2_) and high-frequency band (*fh*_2_). The DC feedline originates from the top layer, passes through the middle layer, and finally enters the DC feedline layer. It is located on the side of the cross-shaped patch, with a 56-nH choke inductor. A quarter-wavelength fan-shaped branch can also effectively suppress the influence of the DC feedline on radio frequency (RF). However, the large size of the fan-shaped branch restricts the scalability of the array. The detailed parameters of meta-atom are provided in Table S1. The relationship between the frequencies mentioned above is *fl*_2_ < *fl*_1_ < *f*_0_ < *fh*_2_ < *fh*_1_. Therefore, the proposed meta-atom exhibits multiple resonant modes in the in-band and out-of-band. By adjusting the state of the PIN diode and the sizes of the structure, 1-bit phase differences can be achieved simultaneously both in-band and out-of-band.

The center frequencies of these 5 bands are *fl*_2c_ = 3.6 GHz, *fl*_1c_ = 4.16 GHz, *f*_0_ = 5.2 GHz, *fh*_2c_ = 6.55 GHz, and *fh*_1c_ = 7.43 GHz, respectively. The structure corresponding to each frequency band is shown in Fig. 2A. The choice of these 4 out-of-band frequencies is determined by the in-band frequency. Our design principle is to achieve out-of-band RCS reduction without increasing the profile height or adding extra RF components, based on the programmable metasurface. Thus, we introduced parasitic structures on the original metasurface. We realized phase gradient through parasitic structures. This allows the utilization of scattering cancellation for out-of-band RCS reduction. After parameter optimization, we added parasitic structures around the in-band structure to introduce low- and high-frequency resonances. The low-frequency to high-frequency ratio, which does not affect the in-band resonance frequency, is approximately 0.8. Therefore, when *fl*_1c_/*f*_0_ = 0.8 and *f*_0_/*fh*_2c_ = 0.8, changes in the parameters of the introduced high- and low-frequency structures do not affect the in-band resonance characteristics. Similarly, changes in in-band parameters do not affect the out-of-band resonance characteristics. Decoupled in-band and out-of-band design is necessary because dynamically regulating the in-band resonance of the programmable metasurface would otherwise affect the out-of-band characteristics. Moreover, to increase the out-of-band bandwidth, new out-of-band high- and low-frequency parasitic structures were introduced, based on the existing ones. The newly introduced parasitic structures couple with the existing ones, creating a new resonance frequency. Specifically, *fl*_2c_/*fl*_1c_ = 0.86 and *fh*_2c_/*fh*_1c_ = 0.88. The coupled-induced resonance points are not adjacent to the original ones, maintaining a certain frequency interval. Thus, the programmable metasurface achieved RCS reduction in 4 out-of-band frequency bands without increasing the profile or adding extra RF components, because of the above design approach. The meta-atom design underwent the processes of Cell-A, Cell-B, and Cell-C, as shown in Fig. 2B. These are associated with the in-band structure, the out-of-band structure, and the final structural design process. Figure 2C to E depicts the working states of Cell-A, Cell-B, and Cell-C. The characteristics of Cell-A, Cell-B, and Cell-C are described below.

**Fig. 3.**
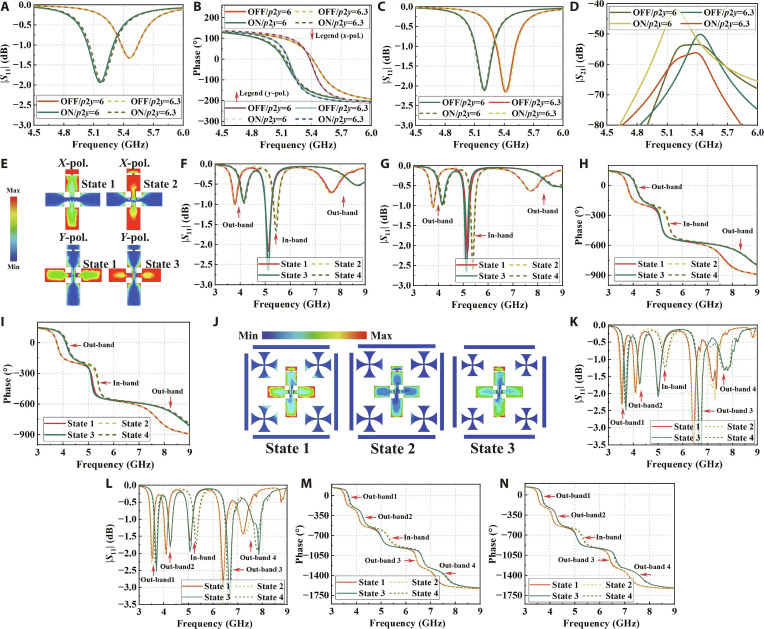
Meta-atom performance. Cell-A’s reflection coefficient for (A) *x*-polarization and (B) *y*-polarization. (C) Cell-A’s phases for *x*-polarization and *y*-polarization. (D) Isolation between *x*-polarization and *y*-polarization in Cell-A. (E) Cell-A’s electric field distribution. Cell-B’s reflection coefficients in (F) *x*-polarization and (G) *y*-polarization. Cell-B’s phases in (H) *x*-polarization and (I) *y*-polarization. (J) Cell-B’s electric field distribution. Cell-C’s reflection coefficients in (K) *x*-polarization and (L) *y*-polarization. Cell-C’s phases in (M) *x*-polarization and (N) *y*-polarization.

The in-band component features a cross structure with 2 separate branches, shown in Cell-A of Fig. 2B. The in-band design follows 2 key principles. First, adjusting the PIN state enables 1-bit phase control for *x*-polarization while maintaining *y*-polarization resonance. Second, variations in the *y*-polarized structure’s size do not influence *x*-polarization. The design principle of Cell-A is described in Note S2. To verify the cross structure’s characteristics, full-wave simulations with periodic boundary conditions were performed. Cell-A parameters are provided in Table S2. The PIN diode was modeled using the equivalent circuit in Fig. 2A (*R* = 0.85 Ω, *L* = 0.45 nH, *C* = 0.21 pF). For *x*-polarization, switching the PIN diode’s ON/OFF states shifts the meta-atoms’ resonance frequencies (Fig. 3A and B), achieving a 1-bit phase difference (180 ± 20°) at 5.2 to 5.4 GHz. Notably, varying *p*2*y* (6 and 6.3 mm) minimally affects reflection coefficients and phases in both PIN states, confirming good *x*-/*y*-polarization isolation. Under *y*-polarization, the meta-atom achieves a 1-bit phase response at 5.25 to 5.4 GHz (Fig. 3B and C), with reflection characteristics closely matching *x*-polarization except for slightly broader *x*-polarized bandwidth. PIN switching does not alter *y*-polarization performance. The cross-polarization isolation is below −40 dB across all 4 states (Fig. 3D). Figure 3E illustrates the electric field distributions: In states 1 and 2, *y*-polarized structures exhibit weak fields under *x*-polarized excitation, while states 1 and 3 show minimal *x*-polarized fields under *y*-polarized excitation.

Traditional out-of-band stealth approaches typically use absorbing frequency-selective surfaces, which may reduce gain and increase profile height. To address these issues, we implement scattering cancellation. To achieve out-of-band stealth separately for high and low frequencies, we introduce high- and low-frequency resonant structures based on Cell-A. These resonant structures, referred to as Cell-B, are designed in a way that they do not affect the in-band characteristics. Cell-B’s structure, shown in Fig. 2B, integrates the original cross with 4 cross-shaped dumbbells and 4 metal branches. The dumbbells enable high-frequency resonance, while the branches facilitate low-frequency resonance. Adjusting *R*1*a* (dumbbell) and *pl* (branches) independently tunes the high- and low-frequency ranges. Design principles and parameters for Cell-B are detailed in Note S3 and Table S3, respectively. Scattering cancellation requires Cell-B to exhibit 1-bit phase responses across sizes and states. Figure 3F to I shows reflection coefficients and phases of Cell-B under different states. Figure 3J displays in-band electric field distributions for states 1 to 3, revealing minimal field intensity on out-of-band structures during in-band excitation. This weak coupling, further validated in Note S3, enables independent phase control for in-band and out-of-band operations.

Cell-B enables independent reflection phase control in in-band and out-of-band regions. However, introducing out-of-band high-/low-frequency structures increases in-band reflection loss (Fig. 3F and G) and narrows out-of-band bandwidth. To address this, Cell-C (Fig. 2B) enhances out-of-band 1-bit phase bandwidth while reducing in-band loss. Building on Cell-B, Cell-C integrates middle-layer resonant structures: high-frequency cross-shaped dumbbells rotated 45° relative to the top layer, and low-frequency metal branches mirroring the top-layer dimensions. Design principles for Cell-C are detailed in Note S4. Figure 3K to N shows reflection coefficients and phases of Cell-C under different states. Cell-C features dual out-of-band resonances (low/high-frequency) and a single in-band resonance. Adjusting Cell-C’s high-/low-frequency dimensions achieve dual-polarized 1-bit phases across 4 out-of-band bands. Tuning the PIN diode and p2y parameters enables in-band 1-bit operation. The detailed performances are provided in Note S5. Five frequency bands maintain *x*-/*y*-polarization phase differences of 180° ± 37° (Table 1). To further enhance the bandwidth of Cell-C, the design methodology is detailed in Notes S6 and S7. This approach involves integrating a dielectric coating onto Cell-C, which substantially improves the 1-bit bandwidth in both the out-of-band and in-band regions, as shown in Figs. S12 and S13. The angular stability in the out-of-band high frequency region is also greatly enhanced, showing minimal variation in bandwidth across different incidence angles. Ultimately, an ultra-wideband characteristic is realized, covering approximately 3.6 to 7.47 GHz (70%), with only a few frequency points slightly deviating from the required 1-bit phase difference. Moreover, despite achieving a nearly continuous ultra-wideband that spans both in-band and out-of-band frequencies, the characteristic frequency ratios *fl*_c_/*f*_0_ ≈ 0.8 and *f*_0_/*fh*_c_ ≈ 0.8 are still maintained. In practical applications, a protective cover is also necessary for the metasurface to prevent performance degradation of the PIN diodes and oxidation of the printed circuit board (PCB). Consequently, this protective cover can be implemented as a dielectric coating. This configuration not only safeguards the device but also greatly enhances the bandwidth. This method represents an optimal, concise, and cost-effective strategy for bandwidth extension. Thereby, the meta-atom design becomes more suited to the broad bandwidth requirements.

**Table 1.** One-bit phase bandwidth of meta-atom

Polarization	Out-of-band1	Out-of-band2	In-band	Out-of-band3	Out-of-band4
*X*-polarization	3.55–3.75	4.05–4.3	5.05–5.35	6.5–6.8	7.25–7.7
*Y*-polarization	3.55–3.75	4.05–4.3	5.1–5.3	6.5–6.8	7.25–7.7

### Design principle and parameter analysis

**Fig. 4.**
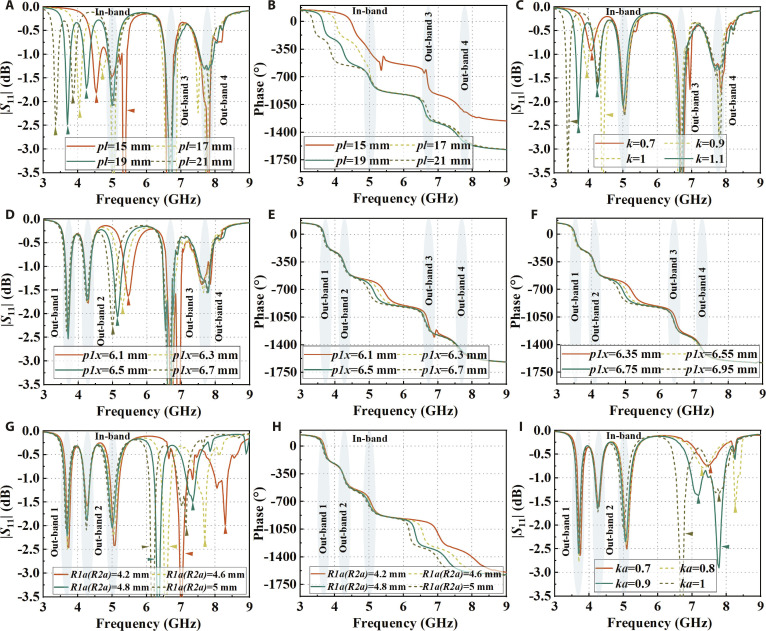
Meta-atom parameter analysis. (A) Reflection coefficient and (B) reflection phase when *pl* changes in state 3. (C) Reflection coefficient when *k* changes in state 3. (D) Reflection coefficient and (E) reflection phase when *p*1*x* changes in state 3. (F) Reflection phase when *p*1*x* changes in state 2. (G) Reflection coefficient and (H) reflection phase when *R*1*a* (*R*2*a*) changes in state 3. (I) Reflection coefficient when *ka* changes in state 3.

To elucidate the selection principle of the 4 frequency bands (out-of-band low-frequency and out-of-band high-frequency), a parameter analysis is subsequently conducted. The coupling characteristics between the parasitic structure and the in-band structure are elaborated. For the proposed meta-atom Cell-C, state 1 and state 3 structures are utilized to achieve out-of-band 1-bit phase modulation. Simultaneously, by modulating the PIN diodes in state 1 and state 3 structures, in-band 1-bit phase modulation can be achieved, corresponding to states 2 and 4, respectively. Consequently, when modulating the PIN diodes on the in-band structure, minimal coupling with the out-of-band parasitic structure is desirable. This avoids the deterioration of the out-of-band 1-bit feature. The key parameters influencing the resonant frequencies of out-of-band low-frequency and out-of-band high-frequency are *pl* and *R*1*a*/*R*2*a*, respectively. First, the coupling characteristics between out-of-band low-frequency and in-band are analyzed. Taking state 3 as an example, the coupling characteristics between in-band and out-of-band are illustrated, with other states exhibiting similar properties. Figure 4A and B presents the reflection coefficient and phase characteristics of the meta-atom when *pl* varies, while other parameters remain constant in state 3. It can be observed that when *pl* varies between 15 and 17 mm, the low-frequency resonant point and the in-band resonant point merge into 2 adjacent resonant points, although the in-band resonant point only experiences a change in reflection amplitude. However, when *pl* varies between 15 and 17 mm, the in-band reflection phase undergoes a significant change. If the *pl* value of the out-of-band low-frequency parasitic structure is set to less than 15 and 17 mm to achieve 1-bit phase modulation, it will inevitably cause the out-of-band low-frequency 1-bit to affect the in-band 1-bit characteristic. Conversely, the significant coupling between the out-of-band low-frequency and the in-band structure prevents independent modulation. Notably, when *pl* varies between 19 and 21 mm, the in-band reflection coefficient and phase remain almost unaffected, with the in-band reflection phases nearly overlapping. Thus, at this point, the coupling between the in-band structure and the out-of-band low-frequency structure is very weak, enabling independent modulation. Consequently, for Cell-C, the *pl* values corresponding to states 1 and 3 are 19.8 and 19 mm, respectively, to achieve out-of-band low-frequency 1-bit phase modulation. With *fl*_1c_/*f*_0_ = 0.8, a decoupled design between out-of-band low-frequency and in-band is achieved. However, due to the small distance between out-of-band low-frequency and out-of-band high frequency, variations in the out-of-band low-frequency can affect the out-of-band high frequency. Nevertheless, through an integrated out-of-band low- and high-frequency design, considering the coupling characteristics, simultaneous 1-bit phase modulation of out-of-band low and high frequency is achieved. Furthermore, regarding the design principle of the 2 resonant frequencies for out-of-band low frequency, Fig. 4C illustrates the relationship between the 2 low-frequency resonant points with different ratios of the key parameters *plu* and *plm* of the top and middle layers’ low-frequency structures, where *plm*/*plu* = *k*. Here, other parameters of state 3 and *plu* = 19 mm are kept constant. It can be seen that when *k* = 0.7, there is only one resonant point. When *k* exceeds 0.7, 2 resonant points emerge in the out-of-band low frequency. For *k* values of 0.9, 1, and 1.1, *fl*_2_/*fl*_1_ are approximately 0.88, 0.86, and 0.79, respectively. Thus, by selecting different *k* values, the number of resonant frequency points for out-of-band low frequency can be determined.

Figure 4D and E illustrates the effect of varying the in-band structural parameter *p*1*x* on the out-of-band high and low frequencies, while other parameters are held constant in state 3. Here, *p*1*x* is the key parameter influencing the in-band structure. As shown in the previous analysis, in state 3, the out-of-band structural parameter *pl* is 19 mm, and the in-band and out-of-band low-frequency structures are decoupled. From the reflection coefficient and phase, it can also be seen that when *p*1*x* changes, the reflection coefficient and phase of the out-of-band low frequency are almost unaffected. It should be noted that when *p*1*x* varies from 6.1 to 6.7 mm, it affects the first resonant point of the out-of-band high frequency, with minimal impact on the second resonant point. Moreover, when *p*1*x* changes from 6.3 to 6.7 mm, the effect on the second high-frequency resonant point is also negligible. Therefore, by adjusting *p*1*x*, the in-band structural changes can be made without affecting the high-frequency out-of-band structure. Figure 4F also shows the effect of *p*1*x* variation on the out-of-band reflection phase under state 1. Similar conclusions can be drawn. Thus, when *p*1*x* is 6.65 mm in states 1 and 2, the in-band PIN diode modulation has almost no effect on the out-of-band high-frequency characteristics, and /*f*_0_/*fh*_2c_ = 0.8. Furthermore, Fig. 4G and H presents the impact of varying the out-of-band high-frequency structural parameter *R*1*a* (*R*2*a*) on the out-of-band low frequency and in-band, while other parameters are kept constant in state 3. It can be observed that when *R*1*a* (*R*2*a*) varies from 4.2 to 5 mm, it has a slight effect on both the out-of-band low frequency and the in-band. That is, as *R*1*a* (*R*2*a*) increases, the out-of-band high-frequency resonant point shifts toward lower frequencies. Similarly, the in-band and out-of-band low-frequency resonant points also shift toward lower frequencies, but at a much slower rate than the high frequency. Therefore, the structural changes required for the out-of-band high frequency to meet the 1-bit demand result in frequency shifts of the in-band and out-of-band resonant points that are almost negligible. Thus, for state 1, *R*1*a* and *R*2*a* are 4.85 and 4.7 mm, respectively. For state 3, *R*1*a* and *R*2*a* are both 4.5 mm. At this point, the structural changes required for the high frequency to achieve 1-bit have minimal impact on the in-band resonant characteristics. Additionally, regarding the design principle of the 2 resonant frequencies for the out-of-band high frequency, Fig. 4I illustrates the relationship between the 2 low-frequency resonant points with different ratios of the key parameters *R*1*a* and *R*2*a* of the top and middle layers’ high-frequency structures, where *R*2*a*/*R*1*a* = *ka.* Here, other parameters of state 3 and *R*1*a* = 4.5 mm are kept constant. It can be seen that when *ka* = 0.7, there is only one resonant point. When *ka* exceeds 0.7, 2 resonant points emerge in the out-of-band high frequency. For *ka* values of 0.8, 0.9, and 1, *fh*_2_/*fh*_1_ are approximately 0.89, 0.88, and 0.88, respectively. Moreover, although only the parameter analysis for state 3 is provided, similar results are observed for other states. Therefore, through the above parameter analysis, it can be seen that once the in-band structure is determined, the out-of-band high- and low-frequency phase modulation can be achieved via the parasitic structure. Additionally, the condition for independent modulation between in-band and out-of-band structures is approximately *fl*_1c_/*f*_0_ ≈ 0.8 and *f*_0_/*fh*_2c_ ≈ 0.8.

**Fig. 5.**
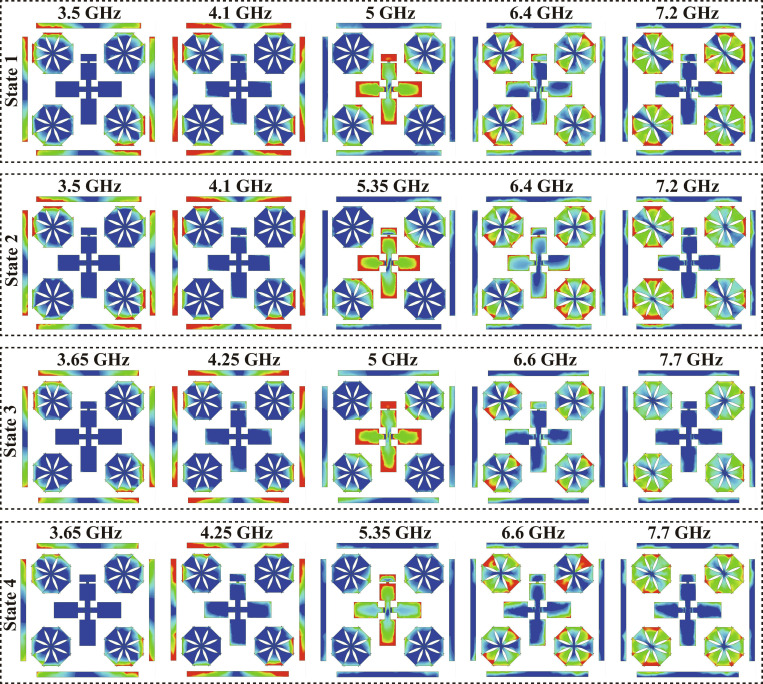
Surface electric field distribution of Cell-C in 4 states.

To further illustrate the coupling characteristics among out-of-band low-frequency, in-band, and out-of-band high-frequency, the meta-atom electric field distribution is given. Figure 5 presents the electric field distributions corresponding to 5 resonant frequencies across 4 states. Notably, when the low-frequency out-of-band structure is excited, the coupling with the in-band structure is minimal. Similarly, when the in-band structure is excited, the coupling with both out-of-band low and high frequencies is also weak. The electric field distributions align with the conclusions drawn from the parameter analysis. Furthermore, when the out-of-band high-frequency structure is excited, there is some coupling at the *f*_*h*2_ frequency point with both the out-of-band low frequency and in-band. However, the coupling at the *f*_*h*1_ frequency point with the in-band structure is relatively small, although the coupling between the *f*_*h*2_ frequency point and the in-band structure may lead to the out-of-band high frequency affecting the in-band characteristics when implementing 1-bit modulation. However, as established in the above parameter analysis, the structural changes required for the out-of-band high frequency to meet the 1-bit demand result in frequency shifts of the in-band and out-of-band resonant points that are almost negligible. Additionally, the out-of-band low and high frequencies can be designed integrally. Thus, following the above design guidelines, it is possible to achieve a low-profile self-stealth programmable metasurface with in-band and out-of-band RCS reduction characteristics. Furthermore, under this design principle, higher-frequency and lower-frequency parasitic structures can be added to further enhance the out-of-band RCS reduction bandwidth. For instance, higher-frequency resonant structures can be incorporated into the vacant regions surrounding the high-frequency parasitic units. Similarly, lower-frequency resonant structures can be introduced into the vacant areas around the low-frequency parasitic units.

### In-band radiation of programmable metasurface

**Fig. 6.**
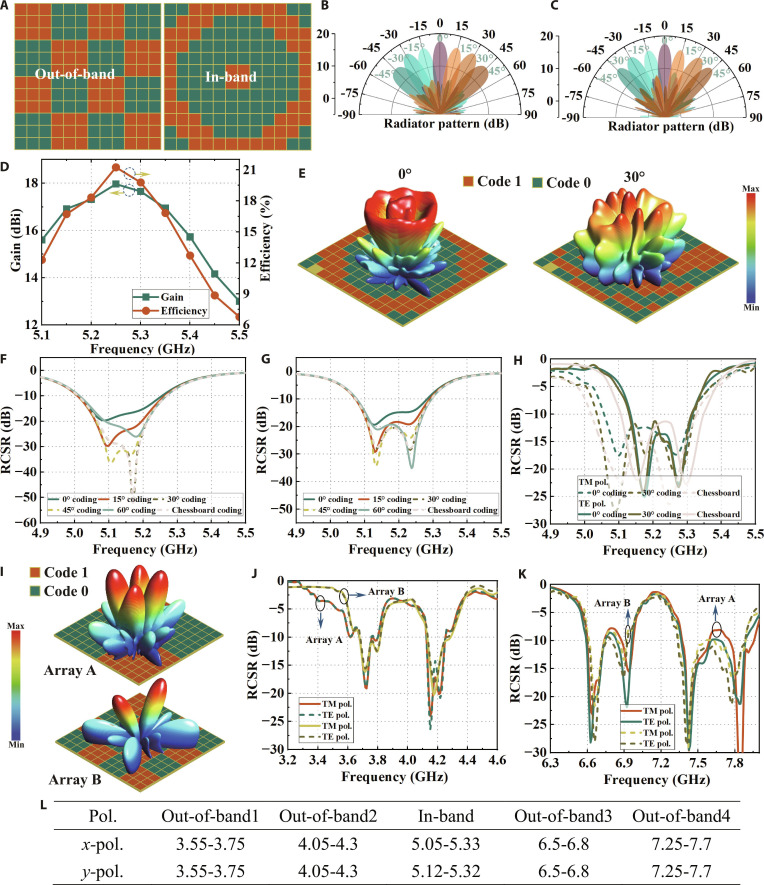
Simulation results. (A) Coding pattern. Beam scanning in (B) E-plane and (C) H-plane. (D) Gain and efficiency. (E) Three-dimensional scatter pattern. Calculated results of RCS reduction in (F) *x*-polarization and (G) *y*-polarization. (H) Simulated RCS reduction results in the in-band. (I) Coding pattern of the array in the out-of-band. Simulated RCS reduction in (J) low-frequency and (K) high-frequency out-of-band. (L) In-band and out-of-band RCS reduction bandwidth.

This study achieves in-band and out-of-band stealth while preserving the programmable metasurface’s in-band radiation performance. Cell-C is arranged in a 12 × 12 array to exhibit the radiation characteristics for *x*-polarization when excited by a horn, as shown in Fig. 1. The out-of-band structures are arranged in a checkerboard topology, as depicted in Fig. 6A. Codes 1 and 2 represent 2 element states with a 1-bit phase difference. The phase distribution of the in-band *x*-polarized structure switches to different encoding patterns based on different beam steering states. For example, when the beam points to 0°, the in-band encoding pattern is depicted in Fig. 6A. The phase encoding pattern for in-band *y*-polarization is consistent with the array beam pointing to 0° as well. By controlling the PIN diodes of the 12 × 12 programmable metasurface, the array can achieve 2-dimensional beam scanning in the E-plane and H-plane. For an array beam deflection angle of (*θ*, *φ*), each element requires continuous compensatory phase:

$$\varphi_{\mathrm{mn}}=\varphi_{f}-\left(\mathrm{kmd}\sin\theta \cos\varphi +\mathrm{knd}\sin\theta \sin\varphi \right)$$ (1)

where *θ* and *φ* represent the elevation and azimuth angles, respectively. *k* is the propagation constant, *d* is the period, and *φ_f_* denotes the phase delay from the feed source to the (*m*, *n*) element. The phase delay caused by the feed source can be expressed as:

$$\varphi_{f}=k\sqrt{{\left(x-x_{0}\right)}^{2}+{\left(y-y_{0}\right)}^{2}+{\left(z-z_{0}\right)}^{2}}$$ (2)

where (*x*, *y*, *z*) represents the position of each element. (*x*_0_, *y*_0_, *z*_0_) is the position of the feed source, where *x*_0_ = 0, *y*_0_ = 0, *z*_0_ = F. The focal diameter ratio (FDR) is defined as *F*/*D*, where *D* is the size of the array. After 1-bit quantization, the required compensatory phase for each unit is given by:

$$\varphi_{\mathrm{mn}}=\left\{\begin{array}{cc} 0^{\circ } & 0^{\circ }\leq \varphi_{\mathrm{mn}}<180^{\circ }\\ 180^{\circ } & 180^{\circ }\leq \varphi_{\mathrm{mn}}<360^{\circ } \end{array}\right.$$ (3)

The discrete phases are encoded as codes 0 and 1, respectively. FDR is 0.75. Figure 6B and C presents the beam scanning results in the E-plane and H-plane, respectively, at 5.2 GHz. It is evident that the programmable metasurface can achieve good 2-dimensional ±45° beam scanning with a scanning step size of 15°. The coding pattern for beam pointing at 15°, 30°, and 45° is shown in Fig. S4. The average sidelobe is below −10 dB, and the cross-polarization is below −30 dB. Figure 6D illustrates the gain and efficiency at different frequencies. It is clearly observed that the 1-dB gain bandwidth is 5.15 to 5.35 GHz. The maximum gain is 17.96 dBi, and the aperture efficiency is 21.25%. Therefore, the programmable metasurface exhibits excellent beam scanning characteristics and high efficiency.

### In-band stealth of programmable metasurface

As previously noted, the programmable metasurface demonstrates excellent *x*-polarization radiation characteristics within the in-band. This section examines its in-band stealth performance during beam scanning. For a 2-dimensional metasurface, the monostatic RCSR can be approximated analytically as follows [52]:

$$\text{RCSR}=10\log10\left({\left|\frac{1}{\mathrm{MN}}\sum_{m=1}^{M}\sum_{n=1}^{N}\left|\Gamma_{\mathrm{mn}}\right|e^{j\varphi_{\mathrm{mn}}}\right|}^{2}\right)$$ (4)

where $\Gamma_{\mathrm{mn}}$ and $\varphi_{\mathrm{mn}}$ are the reflection coefficient and phase of the *mn*th element, respectively. Equation (1) neglects the coupling between the elements and edge effects, but it provides a good guideline to predict the RCS reduction characteristics of the metasurface.

The phase required for beam scanning of the 1-bit programmable metasurface with horn excitation is a combination of code 0 and code 1 elements. For example, the coding pattern of the broadside is shown in Fig. 6E. It is evident that the phase distribution is composed of multiple code 0 and code 1 elements. This indicates that the phase distribution of the array exhibits scattering cancellation for the incident plane wave during beam scanning [53,54]. Based on Eq. (4), the coding patterns of the 1-bit programmable metasurface are calculated for different deflection angles, and their RCS reduction characteristics under these coding patterns are analyzed. The theoretical calculations yield the RCS reduction results under *x*-polarized and *y*-polarized plane wave incidence, as shown in Fig. 6F and G. It is evident that the −10-dB RCS reduction bandwidth is 5 to 5.27 GHz for *x*-polarization and 5.05 to 5.3 GHz for *y*-polarization under different deflection angles. This indicates that the 1-bit programmable metasurface with horn excitation achieves scattering cancellation characteristics for plane wave incidence and thus achieves stealth functionality. Figure 6F and G also demonstrates the RCS reduction characteristics of the array under the chessboard topology. It can be observed that the RCS reduction characteristics of the array under the chessboard topology are nearly identical to the RCS reduction bandwidth and magnitude achieved by the phase topology with horn excitation for beam deflection. Therefore, while maintaining the 1-bit programmable metasurface’s in-band radiation functionality, it also possesses in-band stealth capabilities. Figure 6H presents simulation results of RCS reduction using coding at 0° and 30°. For transverse magnetic (TM)-polarized plane wave incidence, the −10-dB RCS reduction bandwidth is 5.05 to 5.33 GHz. The RCS reduction bandwidths under the 0° and 30° coding patterns are nearly the same, with only slight differences in reduction magnitude. For transverse electric (TE)-polarized plane wave incidence, the −10-dB RCS reduction bandwidth is 5.12 to 5.32 GHz, and the RCS reduction bandwidths and magnitudes under the 0° and 30° coding patterns are almost identical. Figure 6H also illustrates the RCS characteristics under chessboard coding. It can be observed that the bandwidth of *y*-polarized RCSR is greatly improved under chessboard coding, while the effect of *x*-polarization is weaker. Specifically, under chessboard coding, the bandwidth of *y*-polarization RCSR at −10 dB is 5.105 to 5.35 GHz, while that of *x*-polarization is 5.017 to 5.327 GHz. It can be observed that the −10-dB RCSR bandwidth is almost the same in the aforementioned coding schemes. Furthermore, considering that the subsequent *y*-polarization at 0° is also used as a communication transmission mode, the *y*-polarization is selected for 0° coding. Thus, the coding scheme for the *y*-polarization component is unique post-fabrication. Therefore, while maintaining the in-band beam scanning characteristics, the programmable metasurface also exhibits RCS reduction characteristics similar to the array’s chessboard topology under different radiation modes.

### Out-of-band stealth of programmable metasurface

In the previous section, we discussed the stealth characteristics of the programmable metasurface within the in-band. In this section, we will explore its stealth capabilities outside the operating band. To achieve monostatic RCS reduction beyond the operating band, we arrange the proposed Cell-C in a chessboard pattern to achieve scattering field cancellation. We employ 2 chessboard arrangements as shown in Fig. 6I. In these arrangements, code 1 corresponds to state 1 (or state 2) in Fig. 2G, while code 2 corresponds to state 3 (or state 4) in Fig. 2G. Due to the good isolation between the in-band and out-of-band frequencies, using states 1 and 2 as code 1 yields the same effect, while using states 3 and 4 as code 2 also produces the same effect.

Such an arrangement proves to be highly effective in achieving RCS reduction in the out-of-band. Figure 6J and K illustrates the RCS reduction characteristics under the 2 array topologies. It is evident that by arranging the meta-atoms in a chessboard pattern, 4 RCS reduction bands can be achieved in the out-of-band. Moreover, the RCS reduction characteristics are nearly identical for both TE- and TM-polarized plane wave incidence, although there is a slight difference in RCS reduction bandwidth and magnitude between array A and array B. Figure 6L presents the out-of-band −10-dB RCS reduction bands for array B. Therefore, the proposed programmable metasurface demonstrates 4-frequency band RCS reduction characteristics in the out-of-band, in addition to its RCS reduction characteristics in the in-band. Next, we will fabricate the programmable metasurface using the topology of array B to validate its full-band stealth capabilities both in- and out-of-band.

### Experimental

**Fig. 7.**
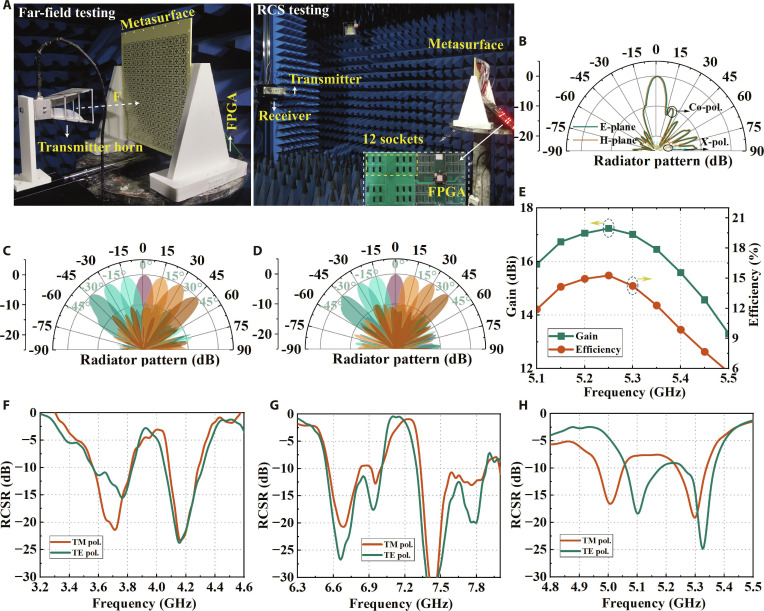
Experimental results. (A) Experiment platform. (B) Far-field radiation patterns in broadside. Beam scanning in (C) E-plane and (D) H-plane. (E) Gain and efficiency. RCS reduction in (F) low-frequency out-of-band, (G) high-frequency out-of-band, and (H) in-band.

To validate the proposed self-stealth programmable metasurface, we fabricated a 12 × 12 programmable metasurface. The layout of the actual processed array is shown in Fig. S15. The top and bottom layers of the processed 12 × 12 array after soldering are shown in Fig. S16. In a microwave anechoic chamber, we conducted tests on the metasurface’s far-field radiation characteristics and monostatic RCS, as illustrated in Fig. 7A. For the far-field radiation characteristics test, we used a transmitter horn to stimulate the metasurface at a position with an *F*/*D* ratio of 0.75. The metasurface’s coding sequence was controlled by an FPGA. In the far-field, we employed a receiver horn to capture the radiated field of the metasurface, thus testing its radiation characteristics. In the RCS test, a transmitter and receiver horn were positioned in the far field to measure the monostatic RCS.

Figure 7B confirms that the proposed metasurface exhibits a high isolation between the co- and cross-polarization. Figure 7C and D displays the beam scanning results in the E-plane and H-plane at 5.2 GHz. It is evident that the metasurface achieves 2-dimensional ±45° beam scanning. The average sidelobe level is below −10 dB. Figure 7E illustrates the gain and efficiency at different frequencies. It can be observed that the 1-dB gain bandwidth is 5.11 to 5.36 GHz, with a maximum gain of 17.23 dBi and an aperture efficiency of 18%. The simulation and measured results are consistent. Therefore, the proposed metasurface demonstrates excellent beam scanning characteristics and efficiency.

During the monostatic RCS test, the programmable metasurface coding is depicted in Fig. 6A, which includes in-band *x-* and *y*-polarizations, as well as an out-of-band coding topology. The measured results demonstrate the RCS reduction characteristics of the metasurface in both the in-band and out-of-band, as shown in Fig. 7F to H. It can be observed that the metasurface exhibits RCS reduction in 4 frequency bands in the out-of-band. The first low-frequency band with a −10-dB RCS reduction extends from 3.57 to 3.82 GHz, and the second low-frequency band from 4.07 to 4.3 GHz. The first high-frequency band is from 6.56 to 7 GHz, and the second high-frequency band is from 7.3 to 7.87 GHz. In the in-band, the −6-dB RCS reduction bandwidth for *x*-polarization ranges from 4.9 to 5.3 GHz, while for *y*-polarization, it spans from 5 to 5.38 GHz. The detailed RCSR measured bandwidth is shown in Table 2. The measured in-band RCS reduction was only −6 dB, with a more severe degradation observed for *x*-polarization. The discrepancies described above are primarily attributable to the following factors. First, the implementation for *x*-polarization required the soldering of PIN diodes and choke inductors. Consequently, this introduced greater uncontrolled errors compared to other polarizations and frequency bands. Thus, soldering inaccuracies for the PIN diodes and choke inductors resulted in a frequency shift of the 1-bit phase bandwidth for *x*-polarization. This led to an in-band RCS reduction for *x*-polarization of less than −6 dB. Second, a deviation in the dielectric constant of the fabricated PCB substrate from the design value caused the RCS reduction across all frequency bands to be slightly inferior to the simulation results. This is because the dielectric constant error altered the 1-bit phase bandwidth. Third, errors were introduced by the measurement environment. This is a common source of uncertainty inherent to all experimental disciplines. Figure S17 presents the measured RCSR performance of the metasurface under oblique plane-wave incidence. Simulated in-band radiation and RCSR performance of metasurface with the coating for oblique incidence are shown in Figs. S18 and S19. Therefore, the proposed programmable metasurface exhibits monostatic RCS reduction characteristics in both the in-band and out-of-band. Moreover, the transient processes and nonlinear effects of PIN diodes, as well as the spectral leakage under rapid beam switching, are crucial for evaluating the performance of metasurfaces in stealth applications. We have conducted corresponding analyses and incorporated relevant additions and clarifications in Note S13.

**Table 2.** In-band and out-of-band RCSR bandwidth

Polarization	Out-of-band1	Out-of-band2	In-band (−6 dB)	Out-of-band3	Out-of-band4
*X*-polarization	3.57–3.82	4.07–4.3	4.9–5.3	6.56–7	7.3–7.87
*Y*-polarization	3.57–3.82	4.07–4.3	5.0–5.38	6.56–7	7.3–7.87

We have added Table 3, which provides a comparison across various aspects including reconfigurability, stealth methodology, requirement for an additional coating, number of RF components, in-band stealth, out-of-band stealth, stealth bandwidth, profile height, and in-band radiation characteristics. It can be observed that the proposed programmable metasurface achieves dual-polarized stealth for both in-band and out-of-band frequencies while exhibiting the lowest profile height. The number of required RF components is also relatively small. Furthermore, it maintains high radiation efficiency within the operating band. Additionally, existing programmable metasurfaces [18,26,48] primarily focus on in-band RCS reduction, whereas only Ref. [50] and the present work achieve out-of-band RCS reduction. However, compared to designs with higher profile heights, such as those in Refs. [50,53], the achievable RCS reduction bandwidth still requires further improvement. Therefore, Table 3 also includes the performance characteristics of the proposed metasurface when integrated with a dielectric coating. It is evident that incorporating the dielectric coating greatly enhances both the in-band and out-of-band RCS reduction bandwidths. Moreover, the in-band and out-of-band frequencies merge to form an ultra-wideband, ultimately resulting in ultra-wideband RCS reduction performance. The overall profile height remains low at merely 0.14λ.

**Table 3.** Comparison between proposed and previous works

Ref. Type	This work	This work + coating	[18]	[26]	[48]	[50]	[53]
Reconfigurable	Yes	Yes	Yes	Yes	Yes	Yes	No
Stealth method	S	S	S	-	S/A	S/A	S
Additional coating	No	Coating	No	-	No	Absorbing	No
RF device	1PIN + 1I	1PIN + 1I	4PIN	8PIN	1PIN	4PIN + 6I + 4R	No
In-band stealth	Yes	Yes	Yes	-	Yes	Yes	Yes
In-band polarization	Dual	Dual	Dual	-	Single	Dual	Dual
Out-of-band stealth	Yes	Yes	No	No	No	Yes	No
Out-of-band polarization	Dual	Dual	-	-	-	Dual	-
Bandwidth	6.8%/5.5%/ 7.8%/6.5%/7.5%	70.71%	14%	-	68.8%	118.7%	141.8%
Profile	0.065λ	0.14λ	0.085λ	0.074λ	0.17λ	0.51λ	0.38λ
In-band radiation	Yes	Yes	Yes	Yes	Yes	Yes	No
Bits	1-bit	1-bit	1-bit	2-bit	1-bit	1-bit	-
Beam scanning	±45°	±60°	±45°	±60°	±60°	±60°	-
Efficiency	18%	23%	17%	35%	15.4%	22.9 %	-

S, scattering cancellation; A, absorbing; I, inductor; R, resistor

## Conclusion

A low-profile self-stealth programmable metasurface with in-band and out-of-band RCS reduction is proposed. Our design principle is to achieve out-of-band RCS reduction without increasing the profile height or adding extra RF components, based on the programmable metasurface. By cleverly modifying the structure of traditional programmable metasurfaces, we achieve high isolation between the co- and cross-polarization within the in-band, enabling independent control of each polarization. The out-of-band and in-band structures are also independent and individually adjustable. Ultimately, our metasurface consists of 4 out-of-band 1-bit regions, namely, the first and second low-frequency out-of-band regions, as well as the first and second high-frequency out-of-band regions. The in-band comprises a 1-bit region for *x*-polarization adjusted using PIN diodes and a 1-bit region for *y*-polarization with varying structural dimensions. By arranging the out-of-band structure in a checkerboard pattern, we achieve scattering cancellation in the incident plane waves, resulting in out-of-band stealth capability. In the in-band, the *x*-polarization region can achieve 2-dimensional ±45° beam scanning when excited by a near-field horn. Additionally, during the beam scanning process, it exhibits scattering cancellation properties even for incident plane waves. Therefore, our programmable metasurface achieves dual functionality of radiation and stealth in the in-band region. Furthermore, the *y*-polarization region exhibits a radiation beam at 0° and also exhibits scattering cancellation characteristics. This research fills the gap in the study of low-profile self-stealth programmable metasurfaces with in-band and out-of-band RCS reduction capabilities. This work is of great significance for programmable metasurface technology.

## Methods

The *S*-parameter performance of meta-atom, and far-field radiation and scattering properties of the metasurface are computed through full-wave numerical simulations with the finite element method solver in high-frequency simulation software (HFSS) of Ansys Electronics Desktop 2022. During the numerical simulation, the PIN diode and capacitors are simulated with an equivalent circuit. In the ON state, the equivalent circuit of the PIN diode is a series combination of a 0.8-Ω resistor and a 0.45-nH inductor. In the OFF state, it is equivalent to a series combination of a 0.21-pF capacitor and a 0.45-nH inductor. We conducted experiment in the microwave chamber to evaluate the metasurface’s radiation and scattering performance. The measurement in the microwave chamber mainly contains receiving standard horn antenna LB-20180 from INFO company and a vector network analyzer N5244A from KEYSIGH company for far-field beam and RCS evaluation of metasurface. In the far-field measurement, the receiving horn records the outgoing wave intensity from the metasurface. The metasurface is fixed on a rotation table to record the radiation and scattering electrical field with different angles. We recorded the output power from a standard horn antenna as a reference to calculate the pattern.

## Data Availability

All data needed to evaluate the conclusions in the paper are present in the paper and/or the Supplementary Materials.

## Supplementary Materials

Supplementary 1 Notes S1 to S13 Figs. S1 to S21 Tables S1 to S13
