# Supplementary material for: A Low-Profile Self-Stealth Programmable Metasurface with In-Band and Out-of-Band RCS Reduction
Source: Research (Wash D C). 2025 Dec 4;8:1017. doi: 10.34133/research.1017 (PMC13248699; doi:10.34133/research.1017)
Supplement: Supplementary 1 — Notes S1 to S13 Figs. S1 to S21 Tables S1 to S13 [file research.1017.f1.docx]

**Supplementary Material for**

A Low-Profile Self-Stealth Programmable Metasurface with In-band and Out-of-band RCS Reduction

**Authors**

Yajie Mu^1^, Jiaqi Han^1^, Lihao Zhu^1^, Dexiao Xia^1^, Xiangjin Ma^1^, Haixia Liu^1^, and Long Li^1*^

**Affiliations**

^1^Key Laboratory of High-Speed Circuit Design and EMC of Ministry of Education, School of Electronic Engineering, Xidian University, Xi'an 710071, China

* Correspondence should be addressed to Long Li; lilong@mail.xidian.edu.cn

**Supplementary Note 1. Design Details of the Meta-atom**

The detailed parameters of meta-atoms in state 1 are shown in Table S1. The two metalized vias in the *y*-axis direction are designed to introduce the y-polarized structure into the intermediate layer, which is connected to each other through branches with a length of *p3x* and a width of *p3y*. Compared to the x-polarized structure, *p1x* is 6.65 mm, and the maximum value of *p*2*y* in the y-polarized structure is 6.2 mm. This is because the y-polarized structure has undergone vertical vias, introducing additional electrical dimensions. The dumbbell-shaped structure in the middle layer rotates 45° relative to the top layer to suppress unnecessary resonance caused by strong coupling between the middle and top structures. In addition, the detailed design parameters of Cell-A and Cell-B are shown in Tables S2 and S3.

**TABLE S1**

**Geometric Parameters of Cell C in State 1**

| Parameters | Value | Parameters | Value | Parameters | Value |
| --- | --- | --- | --- | --- | --- |
| *p1y* | 3 mm | *R1a* | 4.85 mm | *p3y* | 2.5 mm |
| *p1x* | 6.65 mm | *R1b* | 3.75 mm | *R2a* | 4.7 mm |
| *p2x* | 3 mm | *pl* | 19.8 mm | *R2b* | 3.65 mm |
| *p2y* | 6.2 mm | *p3x* | 1 mm | *Lg* | 23 mm |

**TABLE S2**

**Geometric Parameters of Cell A**

| Parameters | Value | Parameters | Value |
| --- | --- | --- | --- |
| *p1y* | 3 mm | *p2y (state3)* | 6 mm |
| *p1x* | 6.75 mm | *p3x* | 1 mm |
| *p2x* | 3 mm | *p3y* | 2.5 mm |
| *p2y (state1)* | 6.3 mm | *Lg* | 23 |

**TABLE S3**

**Geometric Parameters of Cell B in State 1**

| Parameters | Value | Parameters | Value | Parameters | Value |
| --- | --- | --- | --- | --- | --- |
| *p1y* | 3 mm | *R1a* | 4.85 mm | *p3y* | 2.5 mm |
| *p1x* | 6.65 mm | *R1b* | 3.75 mm | *Lg* | 23 mm |
| *p2x* | 3 mm | *pl* | 19.8 mm |  |  |
| *p2y* | 6.2 mm | *p3x* | 1 mm |  |  |

**Supplementary Note 2. Design Details of Cell-A**

In the conventional cross structure, the x- and y-polarized structures are directly connected at the center position, which results in the resonance frequency of the y-polarization being affected by the PIN diodes switching on the x-polarization. Similarly, the size variation of the y-polarized structure also affects the x-polarization. Given these phenomena, we have employed a bridging design to solve the problem of the direct connection between the x-polarization and y-polarization structures of the cross. Specifically, the branches in the x-polarization are directly connected at the center of the top layer, while the branches in the y-polarization extend through via holes to the middle layer and then directly connect at the center. This design approach avoids the direct connection between the x- and y-polarization structures, thereby improving the isolation between the two polarizations.

**Supplementary Note 3. Design Details of Cell-B**

First, we investigate the mutual influence between the low-frequency and high-frequency structures when the in-band structure and PIN state are fixed, as shown in Figures S1a-b. By observing Figures S1a-b, the following conclusions can be drawn. The variations in the low-frequency structure have no significant effect on the reflection characteristics of the high-frequency structure. The variations in the high-frequency structure do affect the low-frequency. When the high-frequency structure increases in size, the resonant frequency of the low-frequency also shifts towards the lower frequency. This is due to the coupling between the high-frequency and low-frequency structures. This study did not employ a decoupling method but instead analyzed the low-frequency and high-frequency structures while considering the presence of coupling. From Figures S1a-b, it can be observed that when *R*1*a* = 4.85 mm/*pl* = 19.8 mm, the reflection phase at 7.75-8.55 GHz exhibits a phase difference of 180°±37°, compared to the corresponding element with *R*1*a* = 4.5 mm/*pl* = 19 mm. Similarly, the reflection phase in the low-frequency of 3.8-4.1 GHz also shows the same phase difference. Therefore, considering the high-frequency and low-frequency structures as a whole and modifying the structural parameters can avoid the coupling influence. Furthermore, it can be observed that the low-frequency and high-frequency structures have minimal impact on the in-band. Next, we will analyze the coupling characteristics between the in- and out-band-of structures when the in-band structure changes. Coupling characteristics between different states:

a) When the state of the in-band structure changes while the out-of-band structures remain unchanged (state1 and state2), the characteristics of the out-of-band structures are not affected by the in-band structure.

b) When the dimensions of the out-of-band structures change while the in-band structure remains unchanged (PIN diode in the ON, state1, and state3), the characteristics of the in-band structure are not affected by the out-of-band structures.

c) When the state of the out-of-band structures changes while the in-band structure remains unchanged (PIN diode in the OFF, state2, and state4), the characteristics of the in-band structure are not affected by the out-of-band structures.


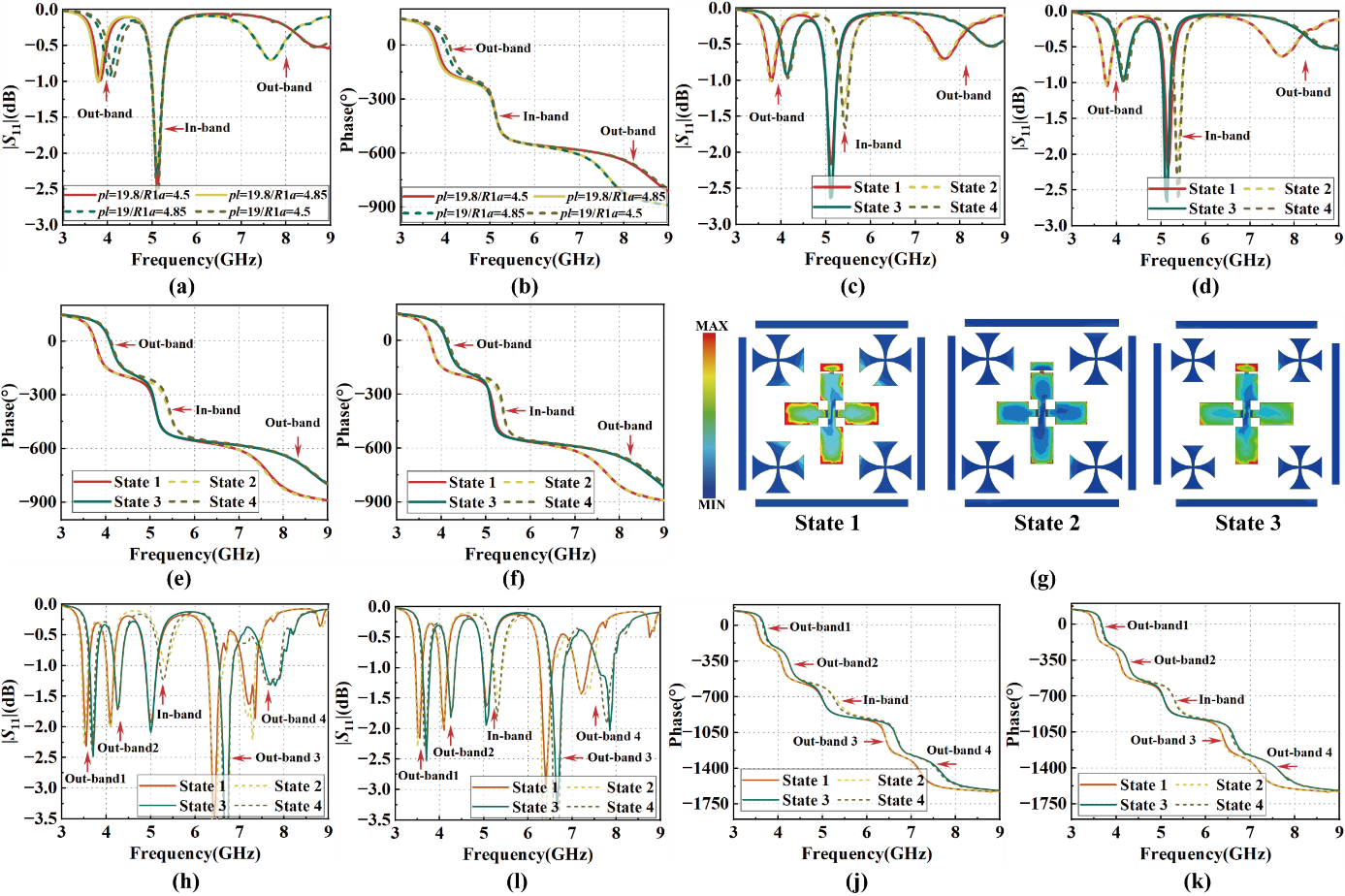


***Figure S1. Meta-atom results.*** *(a) Reflection coefficients and (b) phases of Cell-B when the out-of-band structure parameters are varied.*

**Supplementary Note 4. Design Details of Cell-C**

Based on the analysis in the previous section, there is good isolation between the in-band structure and the out-of-band structures, and the newly introduced high-frequency and low-frequency structures do not affect the in-band. Furthermore, the newly introduced high-frequency and low-frequency structures, along with the original high-frequency and low-frequency structures, can be considered as a unified entity, eliminating the coupling influence. Through this design approach, the low-frequency and high-frequency structures, which originally had a single resonance point, are expanded to two resonance points, further increasing the 1-bit bandwidth in both the low-frequency and high-frequency ranges. It can be observed that the variation in the in-band structure (state 1 and state 2) has minimal impact on the reflection characteristics of the out-of-band structures when the out-of-band structures remain unchanged. Similarly, when the in-band structure remains unchanged and the out-of-band structures undergo changes (state 1 and state 3, or state 2 and state 4), the reflection characteristics of the in-band structure are also minimally affected. By introducing Cell-C, we are able to enhance the 1-bit reflection phase bandwidth of the out-of-band while simultaneously reducing the reflection loss of the in-band. Such a design can further optimize the performance of the metasurface and provide possibilities for broader applications. Furthermore, independent control of x- and y-polarizations in the in-band is achieved. In-band and out-of-band characteristics are independent of each other and do not affect each other.

**Supplementary Note 5. 1-bit Performance in Five Frequency Band**

The reflection coefficients and phases of Cell-C in five frequency bands under x and y polarization are shown in Figures S2 and S3, respectively. The five frequency bands corresponding to an x-polarization phase difference of 180° ± 37° are shown in Table 4S. The five frequency bands corresponding to a y-polarization phase difference of 180° ± 37 ° are shown in Table 5S.


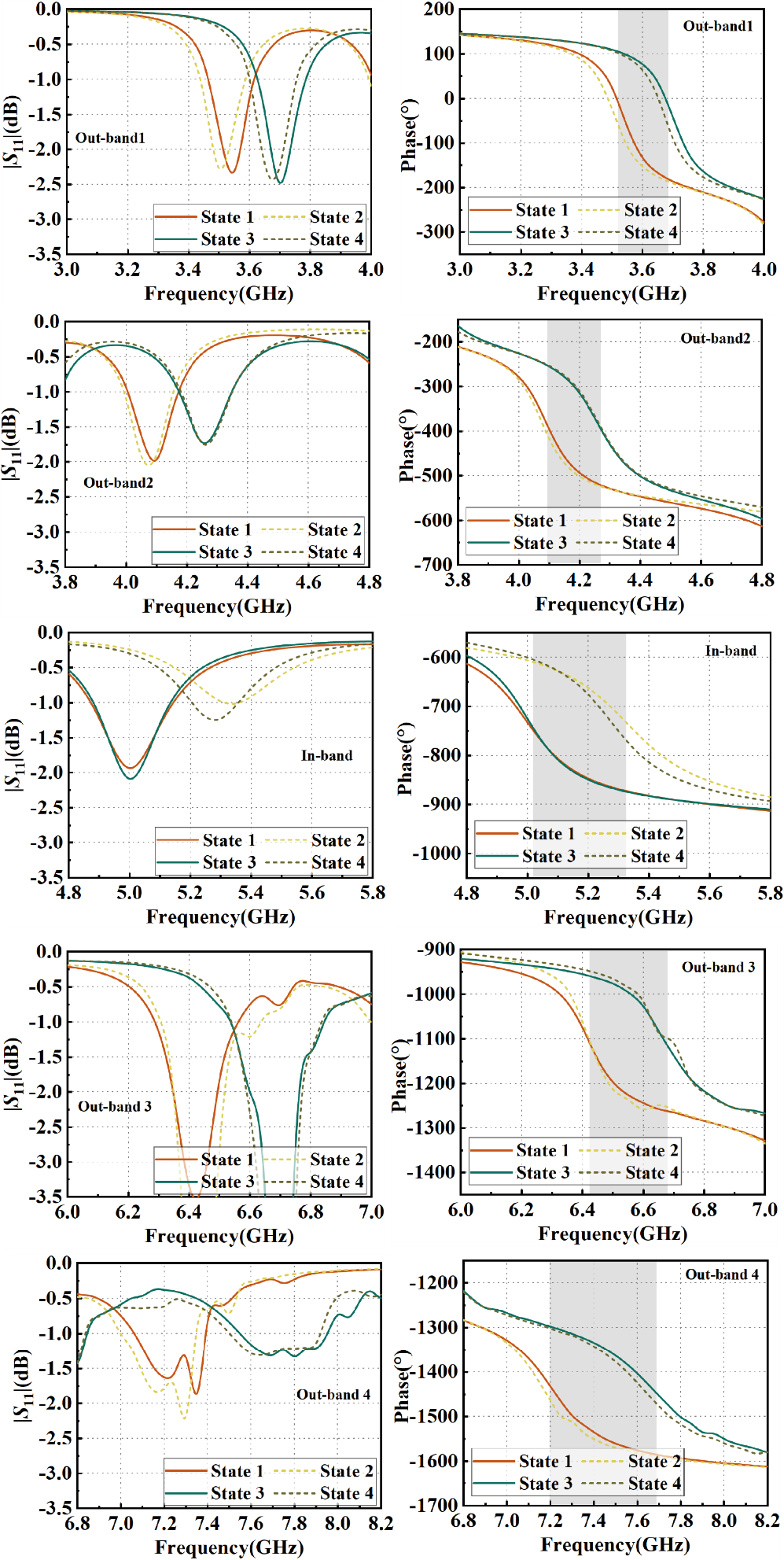


***Figure S2. Detailed reflection coefficient and phase in x-polarization.***


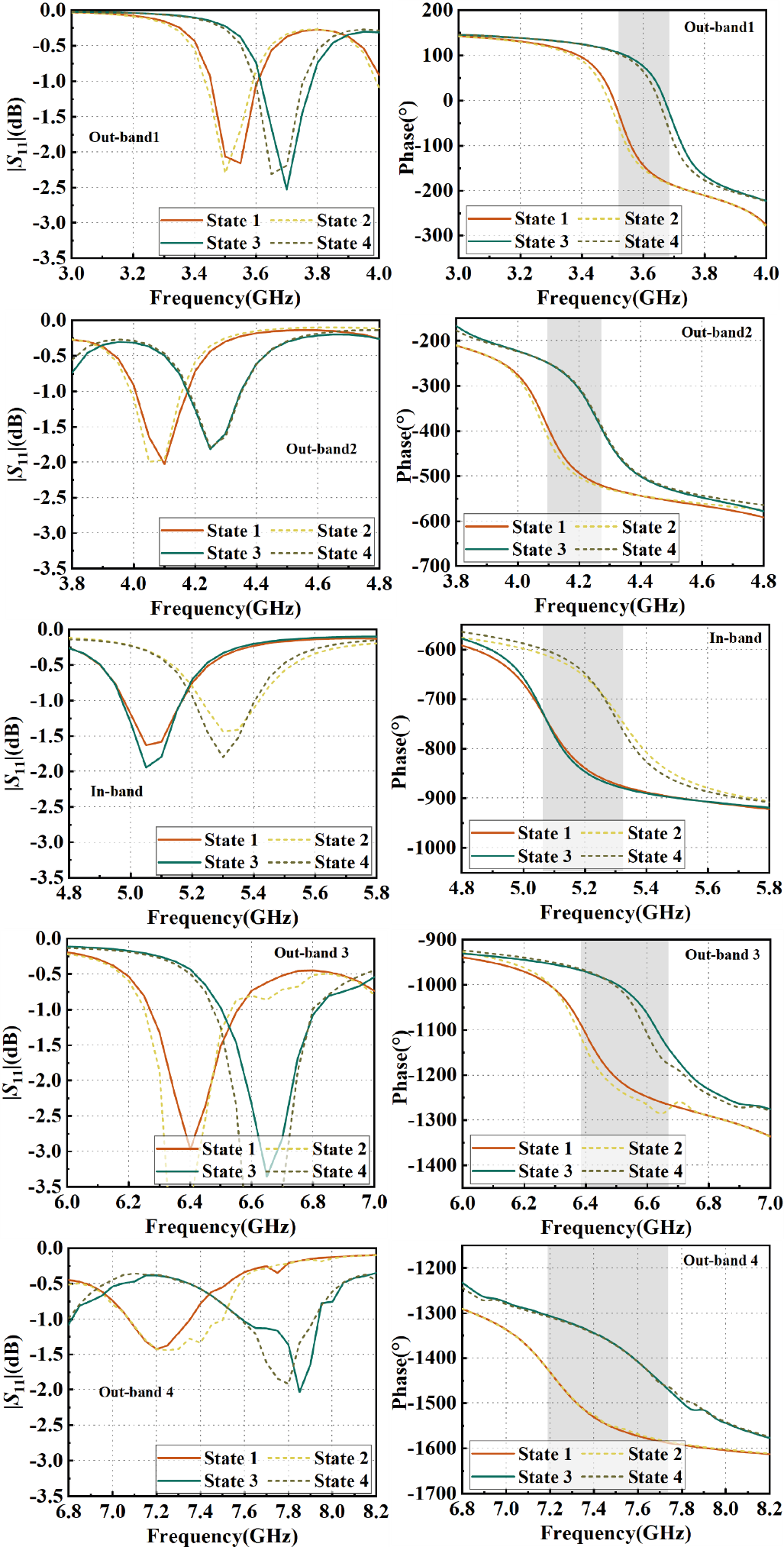


***Figure S3. Detailed reflection coefficient and phase in y-polarization.***

**TABLE S4**

**1-bit bandwidth in *x*-polarized**

| Pol. | Out-of-band1 | Out-of-band2 | In-band | Out-of-band3 | Out-of-band4 |
| --- | --- | --- | --- | --- | --- |
| *x-*pol. | 3.52-3.69 | 4.08-4.27 | 5.02-5.32 | 6.42-6.69 | 7.18-7.70 |

**TABLE S5**

**1-bit bandwidth in *y*-polarized**

| Pol. | Out-of-band1 | Out-of-band2 | In-band | Out-of-band3 | Out-of-band4 |
| --- | --- | --- | --- | --- | --- |
| *y-*pol. | 3.52-3.69 | 4.09-4.27 | 5.06-5.33 | 6.38-6.67 | 7.18-7.73 |

**Supplementary Note 6. Integration of a dielectric coating for enhanced bandwidth**

Two primary methods are employed for bandwidth extension. The first method involves integrating parasitic elements around the in-band structure. Since four out-of-band frequency bands have already been achieved using the aforementioned approach, the available space for adding further parasitic elements without interfering with the in-band performance is limited. This space is only suitable for incorporating high-frequency parasitic elements to realize a fifth out-of-band band beyond *fh*_1_. Moreover, the constrained placement options offer negligible benefits for overall bandwidth enhancement.

The second method utilizes a dielectric coating to flatten the in-band and out-of-band resonances. Consequently, the bandwidth of existing frequency bands is expanded, as demonstrated by Cell-D in Figure S4. A dielectric coating with a thickness of 3.2 mm was integrated 1.5 mm directly above Cell-C. Its dielectric constant is consistent with that of the other dielectric substrates in Cell-C. The reflection characteristics of Cell-D under x-polarization and y-polarization are shown in Figure S5. For comparison, the reflection characteristics of Cell-C are also provided in Figure S6. It can be observed that Cell-D exhibits a substantial improvement in 1-bit bandwidth compared to Cell-C. The x-polarization case is used for illustration. It is evident that Cell-D modifies the frequencies of the two out-of-band resonance points at the lower band. This results in the convergence of the two resonant frequencies, merging them into a single resonance with broader characteristics. The 1-bit bandwidth for the lower out-of-band of Cell-D is approximately 3.6-4.65 GHz, representing a relative bandwidth of 25.4%. In contrast, Cell-C exhibits 1-bit bandwidths of 3.55-3.75 GHz and 4.05-4.3 GHz in the lower out-of-band. Thus, the addition of the dielectric coating to Cell-C enhances the 1-bit bandwidth in the lower out-of-band. Similarly, the in-band 1-bit bandwidth of Cell-D is also improved. Similarly, the in-band 1-bit bandwidth of Cell-D is also improved. The dielectric coating flattens the in-band resonance profile. The in-band 1-bit bandwidth for Cell-D is about 4.95-6.0 GHz, corresponding to a fractional bandwidth of 19.2%. This compares favorably to Cell-C's in-band 1-bit bandwidth of 5.05-5.35 GHz. Thus, the dielectric coating also successfully extends the in-band 1-bit bandwidth. However, the improvement in the upper out-of-band bandwidth for Cell-D is minimal compared to Cell-C. Furthermore, the performance for y-polarization is similar to that of x-polarization, with the exception of some minor differences in the in-band characteristics. The y-polarization 1-bit bandwidth for Cell-D is narrower than for x-polarization, at approximately 4.9-5.5 GHz. Nevertheless, this still constitutes an improvement over the performance of Cell-C. Table S6 presents the detailed 1-bit bandwidth of Cell-D.


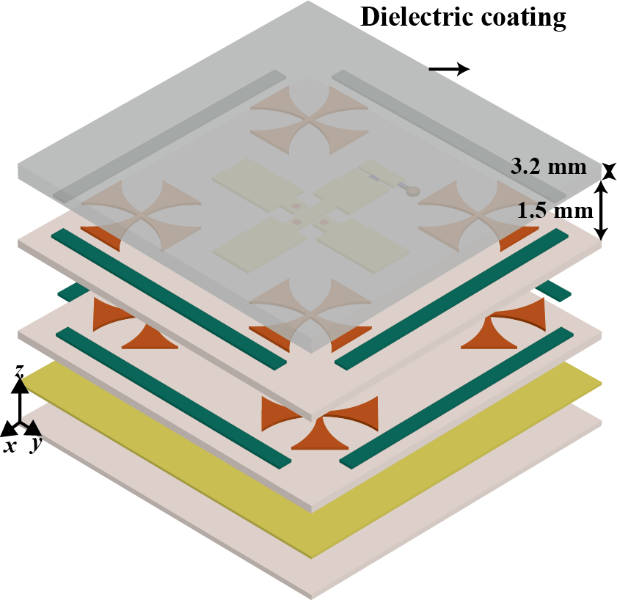


***Figure S4. Integration of a dielectric coating for enhanced bandwidth in meta-atom.***

Consequently, the thickness of Cell-D is increased by 4.7 mm compared to that of Cell-C. The aperture height of Cell-D corresponds to 0.15λ at the in-band frequency (5.2 GHz, consistent with the calculation for Cell-C). Although the profile height more than doubles, a significant improvement in bandwidth is achieved accordingly. Furthermore, in practical applications, a protective cover is also required for the metasurface to prevent PIN diode damage and PCB oxidation. Thus, the protective cover can be designed as a dielectric coating, which not only safeguards the device but also substantially enhances the bandwidth. Essentially, the dielectric protective layer is indispensable for the device. Therefore, this represents an optimal, most concise, and lowest-cost method for bandwidth extension.


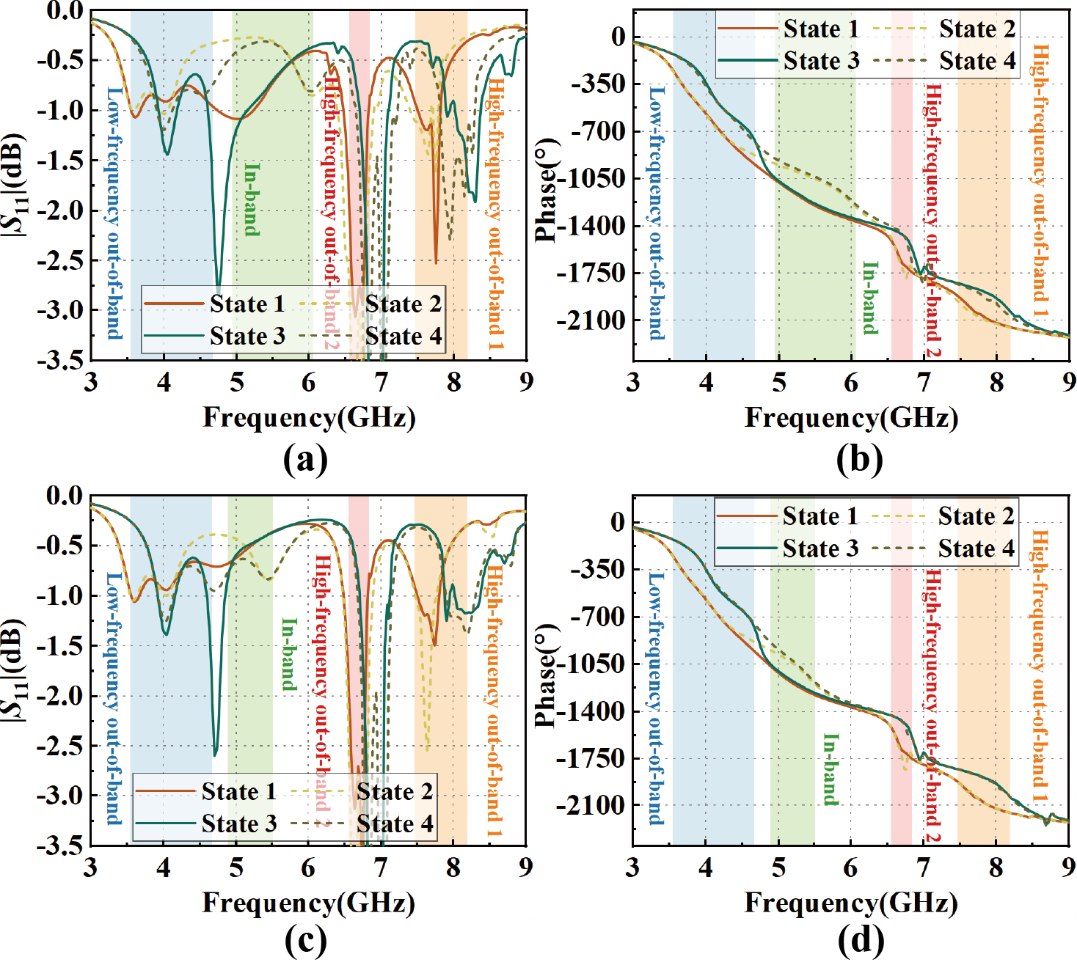


***Figure S5. Integration of a dielectric coating for enhanced bandwidth in meta-atom.*** *Reflection coefficients of (a) x-polarization, and (c) y-polarization. Reflection phases of (b) x-polarization, and (d) y-polarization.*


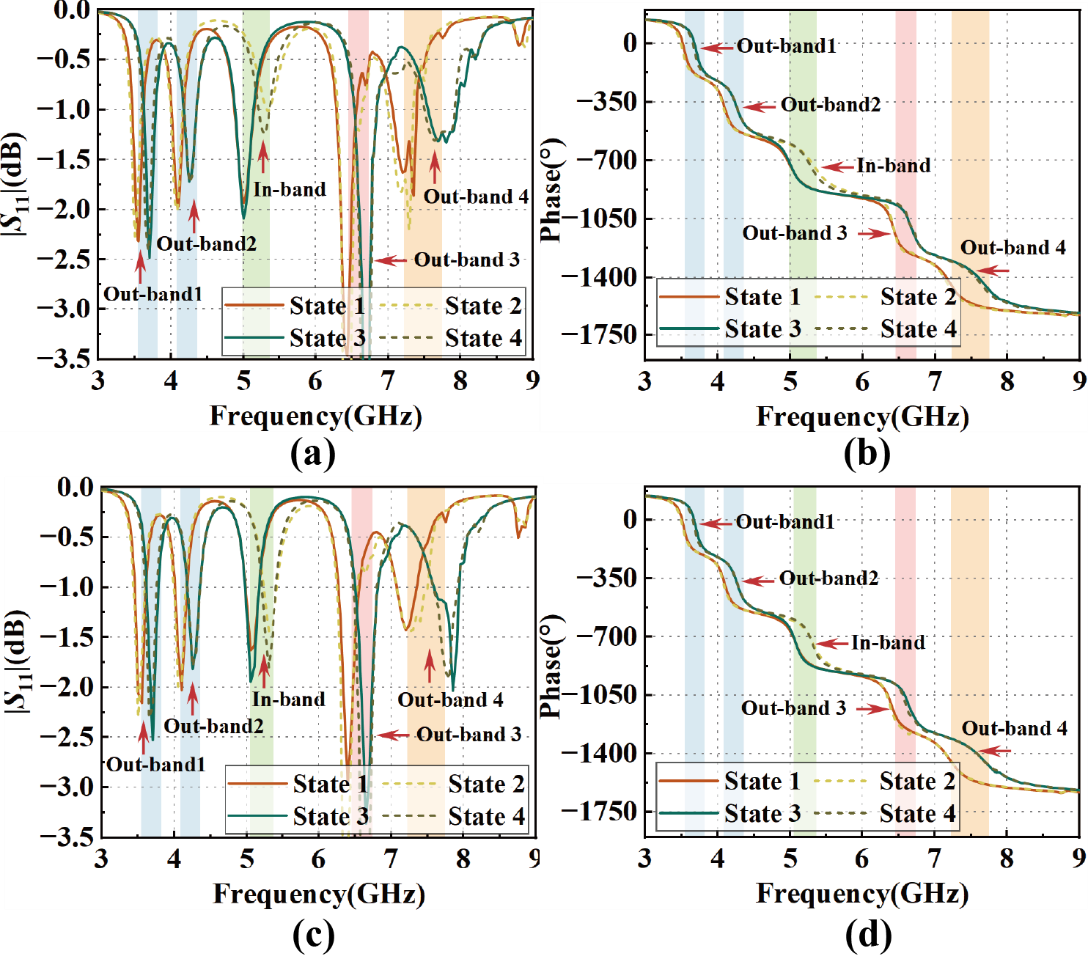


***Figure S6. Origin data of meta-atom.*** *Reflection coefficients of (a) x-polarization, and (c) y-polarization. Reflection phases of (b) x-polarization, and (d) y-polarization.*

**TABLE S6**

**1-bit phase bandwidth of meta-atom with a dielectric coating**

| Pol. | Out-of-band1 | In-band | Out-of-band3 | Out-of-band4 |
| --- | --- | --- | --- | --- |
| *x-*pol. | 3.6-4.65 | 4.95-6.0 | 6.65-6.85 | 7.6-8.1 |
| *y-*pol. | 3.6-3.65 | 4.9-5.5 | 6.65-6.86 | 7.6-8.1 |

**Supplementary Note 7. Analysis of Influencing Factors on Meta-Atom Bandwidth and Angular Stability**

**1. Methods for Enhancing Meta-Atom Bandwidth**

Several primary methods exist for enhancing the bandwidth of a meta-atom: increasing the substrate thickness, using a substrate with a lower permittivity, incorporating parasitic structures, and adding a wide-angle matching layer. Among these, increasing the substrate thickness, incorporating parasitic structures, and adding a wide-angle matching layer are the most effective for bandwidth enhancement. In the proposed design methodology, the incorporation of parasitic structures has already been utilized to achieve multiple out-of-band frequency responses. Consequently, to further enhance the bandwidth of the proposed design, the remaining viable approaches are increasing the substrate thickness and adding a wide-angle matching layer.

**2. Methods for Improving the Angular Stability of Meta-Atoms**

If a meta-atom's reflection characteristics under oblique incidence remain similar to those under normal incidence, its RCS reduction performance can also be maintained at oblique angles. Therefore, the RCS performance can be evaluated by analyzing the degradation of the meta-atom's reflection properties under oblique incidence compared to the normal incidence case. Several principal methods can be employed to improve the wide-angle stability of meta-atoms. Firstly, adopting three-dimensional unit cell structures (transitioning from 2D to 3D) represents the most effective approach for achieving wide-angle stability. This strategy leverages the multi-dimensional resonances provided by 3D structures to average the electromagnetic response across different incidence angles and polarizations. Secondly, utilizing densely packed elements can suppress the emergence of grating lobes, which are a primary cause of performance degradation at large angles of incidence. It is crucial to ensure that no grating lobes appear in the visible space at the highest operational frequency and the maximum incident angle. Alternatively, employing a substrate with a high permittivity is also beneficial. A high-permittivity substrate confines a greater portion of the electromagnetic field within itself, reducing radiation into free space and thereby diminishing sensitivity to the incident angle. Furthermore, using a relatively thin substrate is advantageous for angular stability. Based on the proposed design methodology, the wide-angle stability of the meta-atom can be enhanced through three aspects: adopting a 3D structure, using a high-permittivity substrate, and employing a relatively thin substrate. The proposed design utilizes a dielectric substrate with a permittivity of 3.55, which is already considered high. Additionally, the substrate thickness is 3.5 mm. This substrate is relatively thick for the out-of-band high frequencies, leading to inferior wide-angle RCS performance at these frequencies. However, reducing the substrate thickness would severely compromise the meta-atom's bandwidth. Therefore, improving the wide-angle characteristics must rely on the 3D structure approach. Specifically, employing a wide-angle matching layer can simultaneously enhance both the bandwidth and the wide-angle performance.


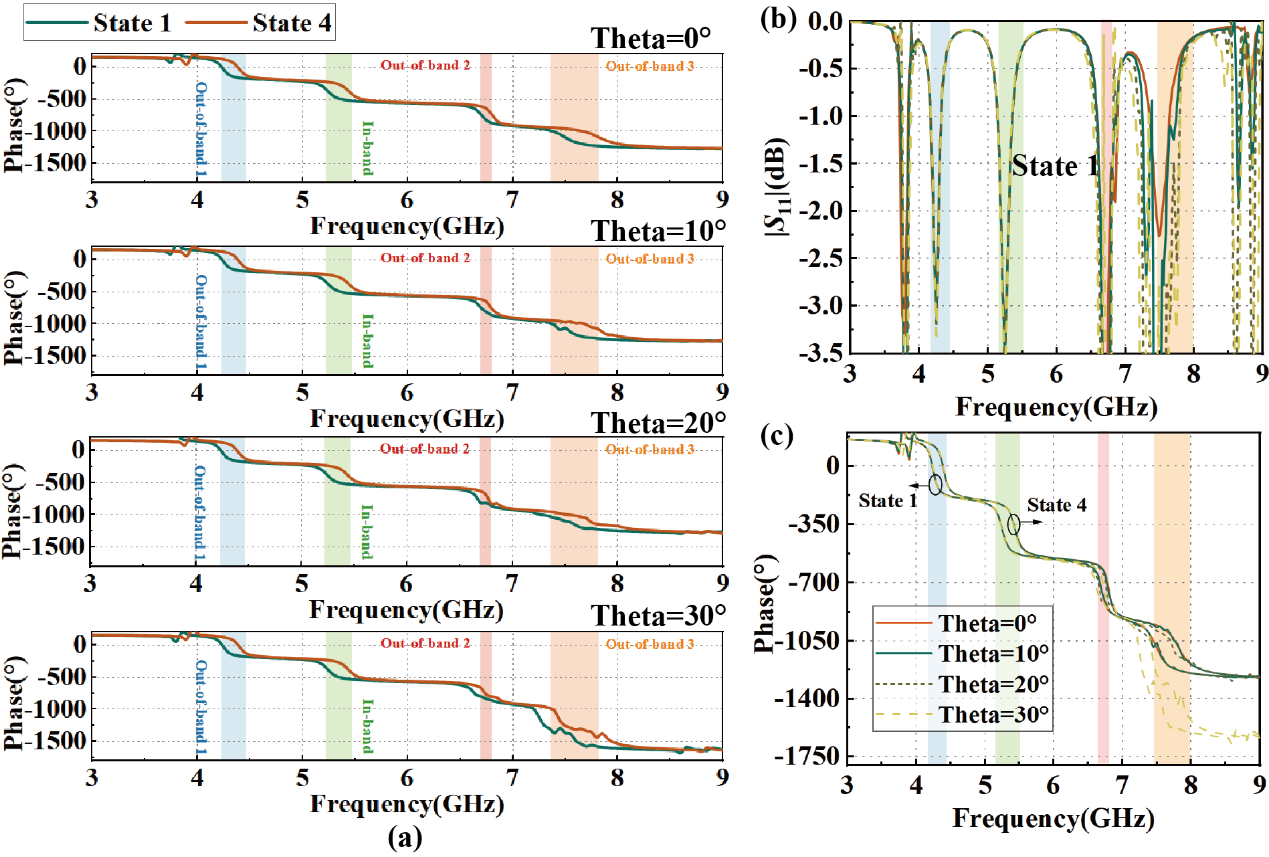


***Figure S7.*** ***Reflection coefficient of meta-atom at different incident angles of x-polarization when substrate thickness is 2.5 mm.*** *(a) Reflection phases at different incident angles are displayed separately. (b) Reflection magnitude under state 1. (c) Reflection phases.*

**TABLE S7**

**1-bit phase bandwidth of meta-atom with substrate thickness is 2.5 mm**

| Theta | Out-of-band1 | In-band | Out-of-band2 | Out-of-band3 |
| --- | --- | --- | --- | --- |
| 0° | 4.25-4.4 | 5.25-5.42 | 6.72-6.79 | 7.53-7.78 |
| 10° | 4.25-4.4 | 5.25-5.42 | 6.71-6.77 | 7.53-7.78 |
| 20° | 4.25-4.4 | 5.25-5.42 | 6.68-6.75 | 7.6-7.7 |
| 30° | 4.25-4.4 | 5.25-5.42 | 6.67-6.7 | 7.57-7.75 |

**3. Simulation Verification**

Based on the preceding analysis, we now present the reflection coefficients under various incidence angles for different substrate thicknesses, dielectric constants, and with the addition of a wide-angle matching layer. This approach concurrently characterizes the bandwidth and angular stability of the meta-atoms. For conciseness and clarity, we present the reflection coefficients only for State 1 and State 4 under x-pol. wave incidence, as States 2 and 3 exhibit similar characteristics.


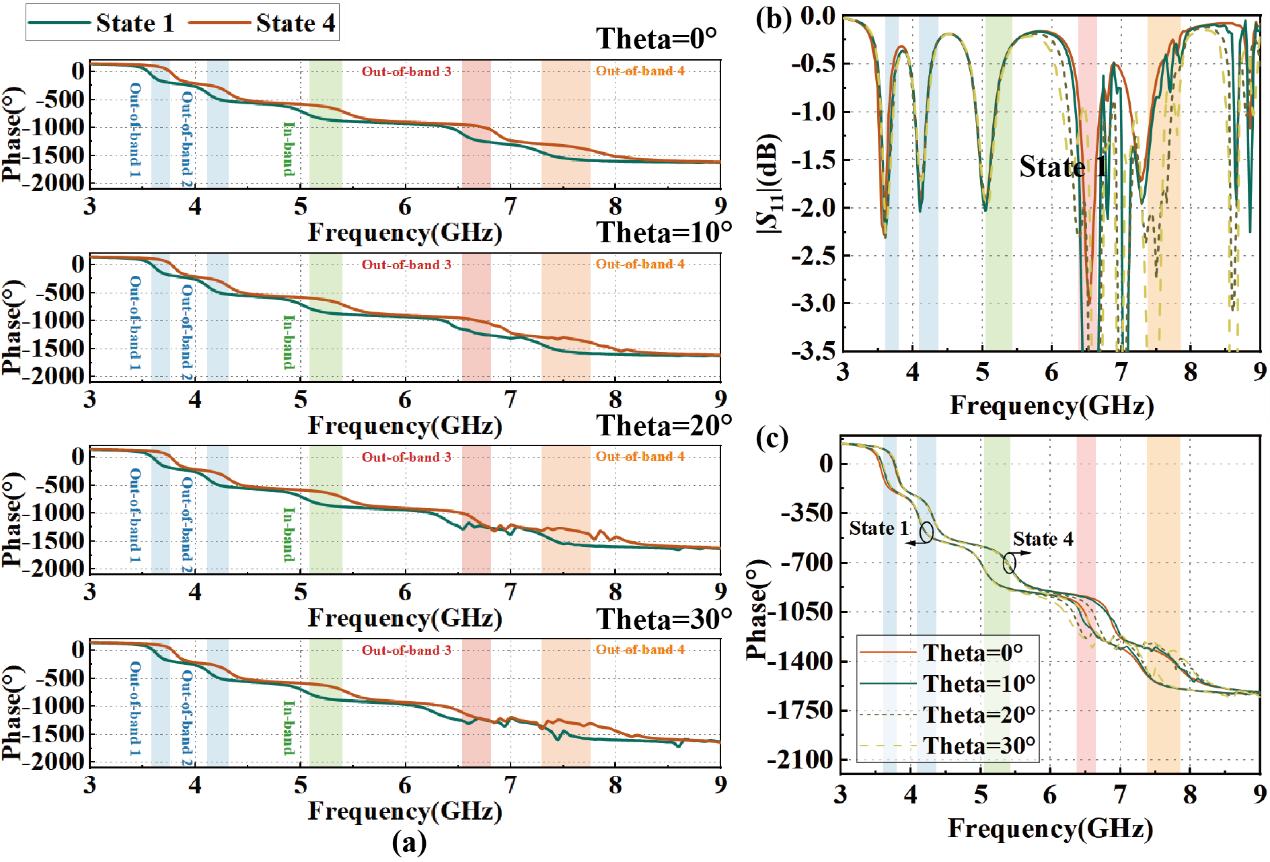


***Figure S8. Reflection coefficient of meta-atom at different incident angles of x-polarization when substrate thickness is 3.5 mm.*** *(a) Reflection phases at different incident angles are displayed separately. (b) Reflection magnitude under state 1. (c) Reflection phases.*

**TABLE S8**

**1-bit phase bandwidth of meta-atom with substrate thickness is 3.5 mm**

| Theta | Out-of-band1 | Out-of-band2 | In-band | Out-of-band3 | Out-of-band4 |
| --- | --- | --- | --- | --- | --- |
| 0° | 3.55-3.75 | 4.05-4.3 | 5.05-5.35 | 6.53-6.87 | 7.25-7.78 |
| 10° | 3.58-3.77 | 4.05-4.3 | 5.05-5.35 | 6.47-6.93 | 7.35-7.83 |
| 20° | 3.58-3.77 | 4.05-4.3 | 5.05-5.35 | 6.37-6.43 | 7.33-7.4 |
| 30° | 3.58-3.77 | 4.05-4.3 | 5.05-5.35 | 6.33-6.53 | 7.37-7.4 |

Figures S7-S9 show the reflection coefficients under different incidence angles for substrate thicknesses of 2.5 mm, 3.5 mm, and 4.5 mm, respectively. Tables S7-S9 provide the corresponding 1-bit phase bandwidths under different incidence angles for substrate thicknesses of 2.5 mm, 3.5 mm, and 4.5 mm, respectively. The 3.5 mm substrate thickness corresponds to the design presented in the manuscript. It is noteworthy that for the 2.5 mm substrate, only one 1-bit frequency band is achieved in the out-of-band low frequency region. In contrast, both the 3.5 mm and 4.5 mm substrates yield two out-of-band low frequency bandwidths. Furthermore, for both in-band and out-of-band frequencies, the bandwidth decreases in the following order: 4.5 mm substrate > 3.5 mm substrate > 2.5 mm substrate. Therefore, increasing the substrate thickness leads to a modest improvement in bandwidth. Another important observation is that the bandwidth characteristics in the out-of-band low frequency and in-band regions remain largely unaffected with increasing incidence angle. However, the out-of-band high frequency performance is sensitive to the incidence angle. This is because a thinner substrate generally confers better angular stability to the unit cell. For a given substrate thickness, the electrical size is larger at higher frequencies. Consequently, the bandwidth at high frequencies narrows under oblique incidence. Accordingly, the angular stability ranks from best to worst as follows: 2.5 mm substrate > 3.5 mm substrate > 4.5 mm substrate. Particularly noteworthy is that for the 4.5 mm substrate, the out-of-band high frequency bandwidth effectively reduces to a few discrete frequencies under oblique incidence. In comparison, for the 2.5 mm substrate, the bandwidth only narrows slightly under oblique incidence. Thus, the substrate thickness directly and significantly influences both the bandwidth and angular stability of the meta-atom. Moreover, the impact of substrate thickness on bandwidth and angular stability is opposing. Therefore, selecting the substrate thickness requires a balanced consideration of both bandwidth and angular stability. Hence, the design in the manuscript adopts an intermediate thickness of 3.5 mm, balancing the 2.5 mm and 4.5 mm cases. It can be observed that the 3.5 mm substrate offers a compromise, with its bandwidth and angular stability lying between those of the 2.5 mm and 4.5 mm substrates.

The above content has already analyzed the influence of the substrate thickness on the bandwidth and the stability of the angle. That all previous examples used a dielectric constant of 3.55. Figure S10 and Table S10 present the reflection coefficients and 1-bit bandwidth for a substrate thickness of 3.5 mm and a relative permittivity of 2.2. It can be seen that compared to the case with *εᵣ* = 3.55, the bandwidth for *εᵣ* = 2.2 is only slightly improved. However, the angular stability in the out-of-band high frequency region is significantly worse for *εᵣ* = 2.2. The angular stability remains relatively good for the out-of-band low frequency and in-band regions. In particular, the two out-of-band high frequency bandwidths degrade into discrete frequencies under oblique incidence. Therefore, a lower substrate permittivity results in poorer angular stability. In contrast, reducing the permittivity has a less pronounced effect on bandwidth. Consequently, selecting a substrate with a higher permittivity is generally preferable for enhancing the angular stability of the meta-atom. The design in the manuscript employs a permittivity of 3.55.


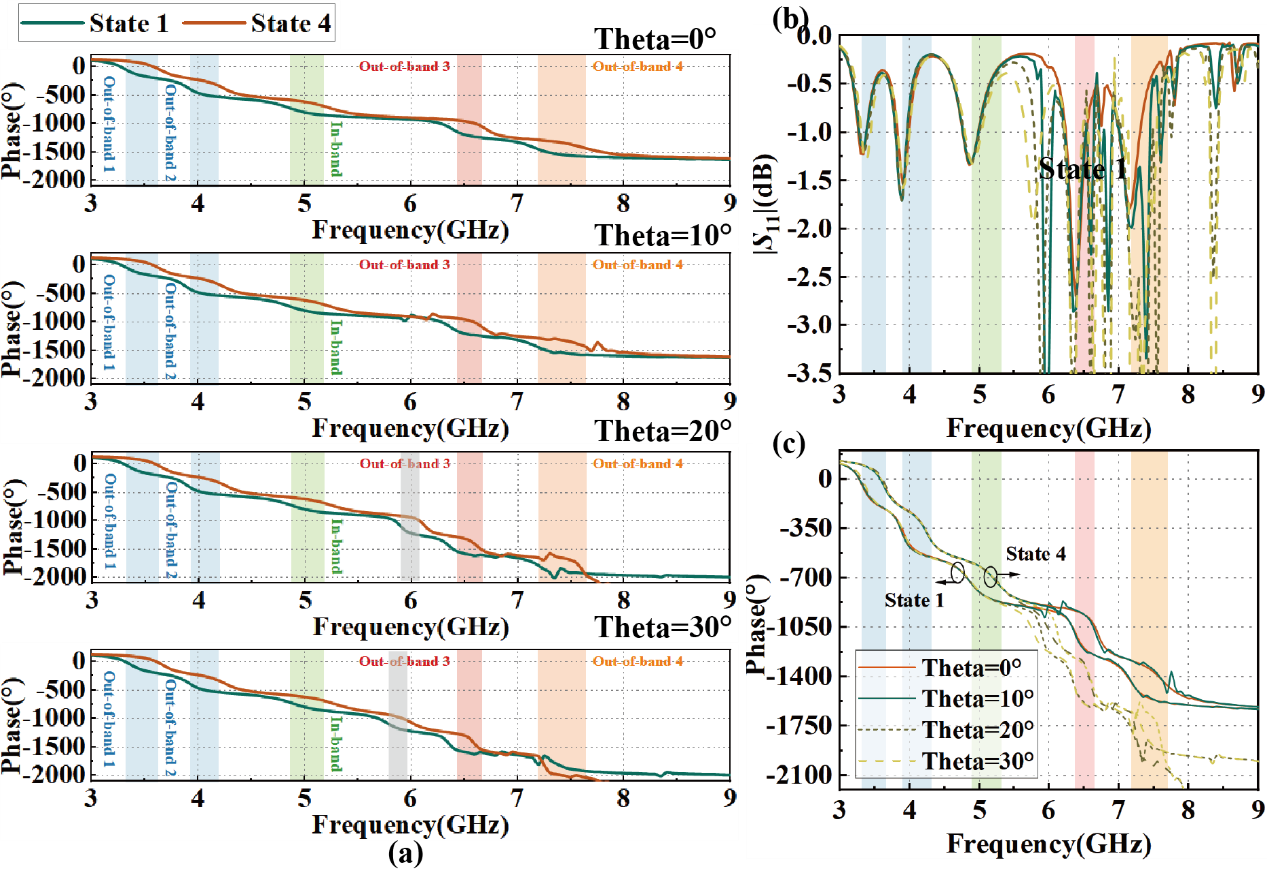


***Figure S9. Reflection coefficient of meta-atom at different incident angles of x-polarization when substrate thickness is 4.5 mm.*** *(a) Reflection phases at different incident angles are displayed separately. (b) Reflection magnitude under state 1. (c) Reflection phases.*

**TABLE S9**

**1-bit phase bandwidth of meta-atom with substrate thickness is 4.5 mm**

| Theta | Out-of-band1 | Out-of-band2 | In-band | Out-of-band3 | Out-of-band4 |
| --- | --- | --- | --- | --- | --- |
| 0° | 3.33-3.65 | 3.87-4.13 | 4.9-5.2 | 6.37-6.7 | 7.18-7.61 |
| 10° | 3.33-3.63 | 3.87-4.13 | 4.9-5.2 | 6.31-6.67 | 7.18-7.62 |
| 20° | 3.33-3.63 | 3.87-4.13 | 4.9-5.2 | 6.34-6.6 | 7.25/7.4/7.55 |
| 30° | 3.37-3.67 | 3.93-4.15 | 4.95-5.23 | 6.35-6.4 | 7.25/7.35-7.41 |


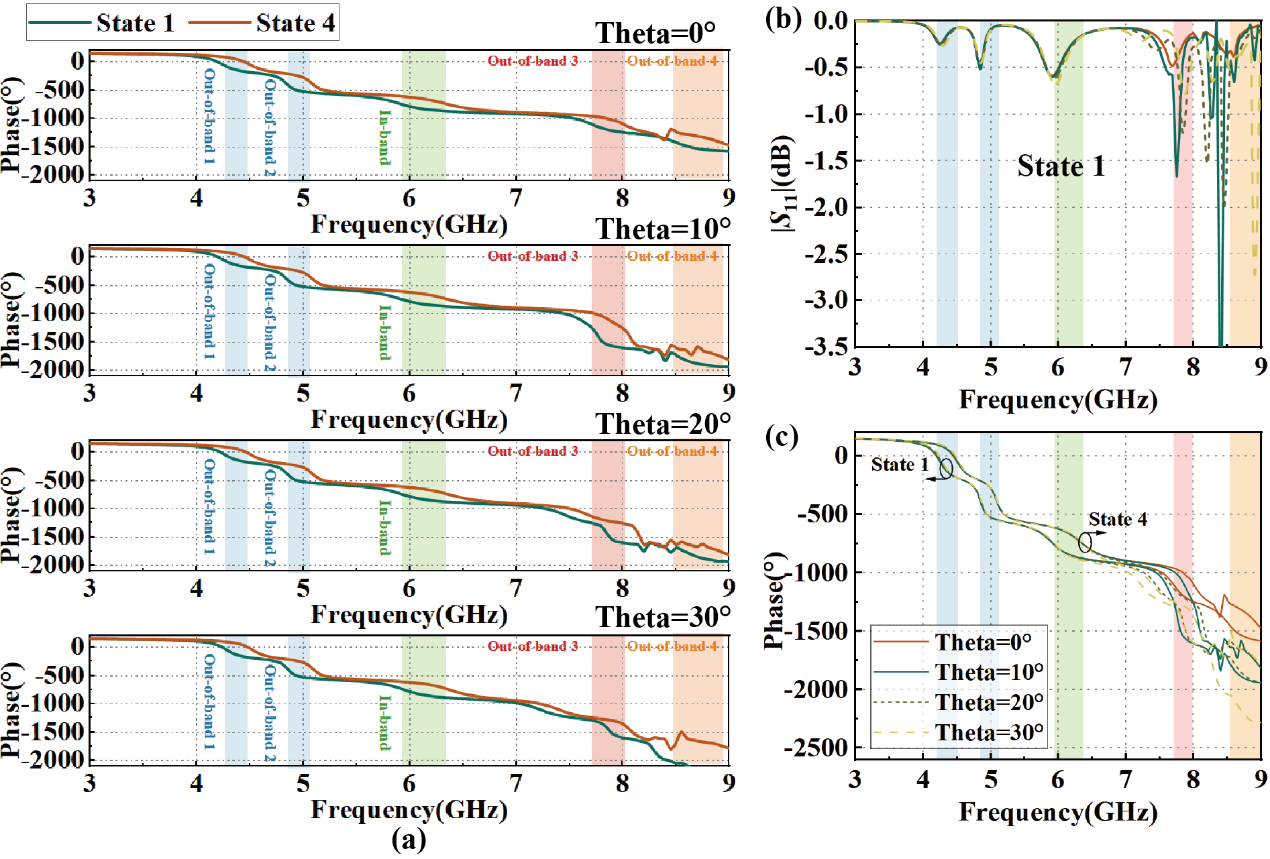


***Figure S10. Reflection coefficient of meta-atom at different incident angles of x-polarization when substrate thickness is 3.5 mm and relative permittivity is 2.2.*** *(a) Reflection phases at different incident angles are displayed separately. (b) Reflection magnitude under state 1. (c) Reflection phases.*

**TABLE S10**

**1-bit phase bandwidth of meta-atom with relative permittivity is 2.2**

| Theta | Out-of-band1 | Out-of-band2 | In-band | Out-of-band3 | Out-of-band4 |
| --- | --- | --- | --- | --- | --- |
| 0° | 4.3-4.45 | 4.85-5.05 | 6-6.25 | 7.77-7.97 | 8.45-8.63 |
| 10° | 4.3-4.45 | 4.85-5.05 | 6-6.25 | 7.65 | 8.55-8.6 |
| 20° | 4.3-4.45 | 4.85-5.05 | 6-6.25 | 7.55-7.6 | 8.55-8.7 |
| 30° | 4.3-4.45 | 4.85-5.05 | 6-6.3 | 7.85 | 8.7-8.9 |

The final method for enhancing both bandwidth and angular stability is the use of a dielectric coating (wide-angle matching layer). Adding a dielectric coating above the unit cell effectively transforms the 2D structure into a 3D one, thereby improving its angular stability. Furthermore, the dielectric coating acts as a form of wide-angle matching layer, which can significantly enhance the bandwidth. Figure S11 and Table S11 present the reflection coefficients and bandwidth results for the case with a 3.2 mm dielectric coating (corresponding to the design in Supporting Information Note 6). The coating is positioned 1.5 mm above the meta-atom. It is evident that the bandwidth is significantly enhanced in both the out-of-band and in-band regions. The two out-of-band low frequency bandwidths merge into a single, broader band. Additionally, the angular stability in the out-of-band high frequency region is markedly improved. The variation in the out-of-band high frequency bandwidth across different incidence angles is relatively small. Furthermore, by optimizing the coating-to-meta-atom distance to 1 mm and engineering the out-of-band high frequency response to form a single wideband characteristic (merging two resonances), an ultra-wideband performance can be achieved. Figure S12 and Table S12 show the reflection coefficients and bandwidth results for a 3.2 mm dielectric coating positioned 1 mm above the meta-atom. It can be observed that the two resonance points in both the low and high out-of-band regions merge into unified wide bandwidths. The angular stability remains good for the low out-of-band and in-band regions. The angular stability in the out-of-band high frequency region is also significantly enhanced, showing minimal variation in bandwidth across different incidence angles. Ultimately, an ultra-wideband characteristic is realized, covering approximately 3.6-7.47 GHz (70%), with only a few frequency points slightly deviating from the required 1-bit phase difference. The reflection coefficients and bandwidths corresponding to y-pol. States 1 and 4 are shown in Figure S13 and Table S13. It can be seen that 1-bit bandwidth is satisfied within 3.6-7.45 GHz, except for 5.9-6.1 GHz.

Moreover, despite achieving a nearly continuous ultra-wideband that spans both in-band and out-of-band frequencies, the characteristic frequency ratios *fl*_c_/*f*_0_ ≈ 0.8 and *f*_0_/*fh*_c_ ≈ 0.8 are still maintained.


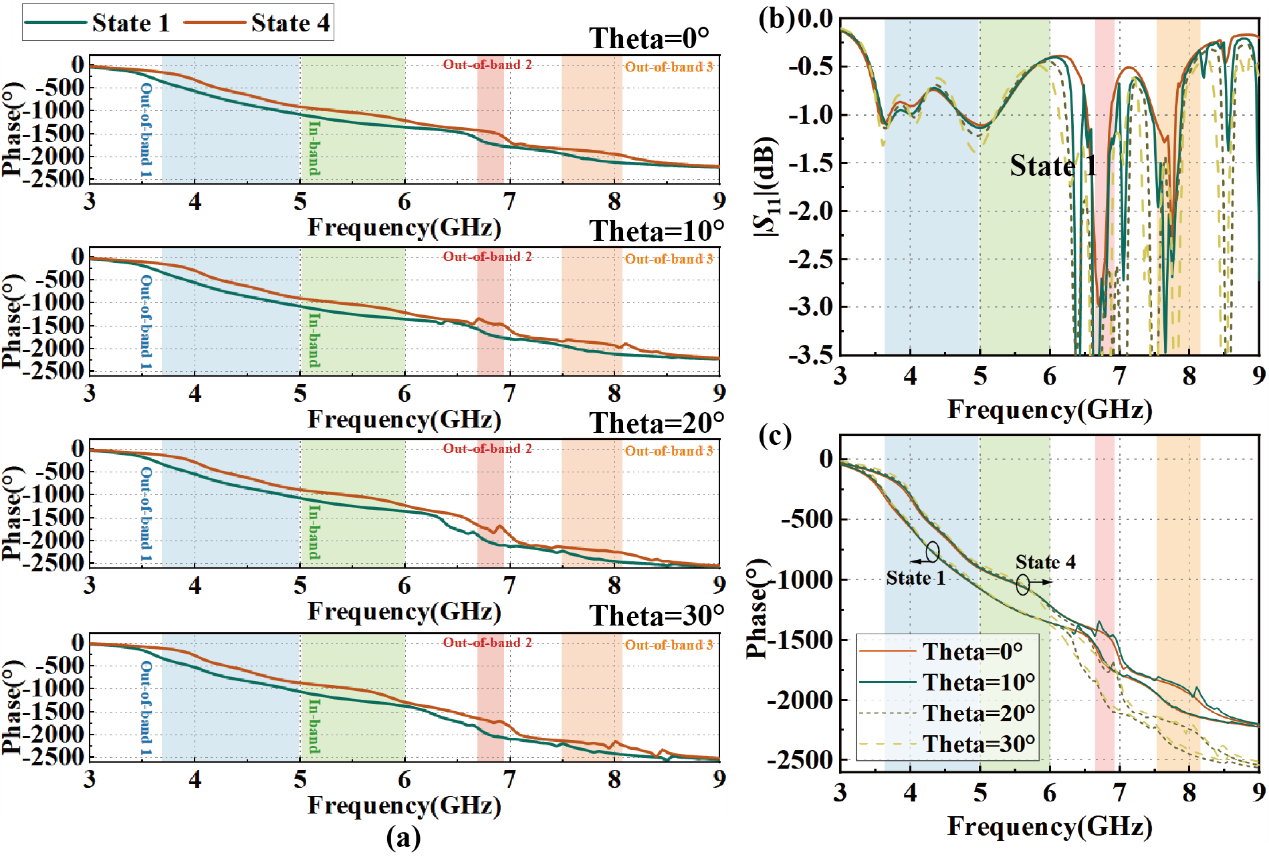


***Figure S11. Reflection coefficient of meta-atom with coating at different incident angles of x-polarization when the coating is 1.5 mm away from the meta-atom.*** *(a) Reflection phases at different incident angles are displayed separately. (b) Reflection magnitude under state 1. (c) Reflection phases.*


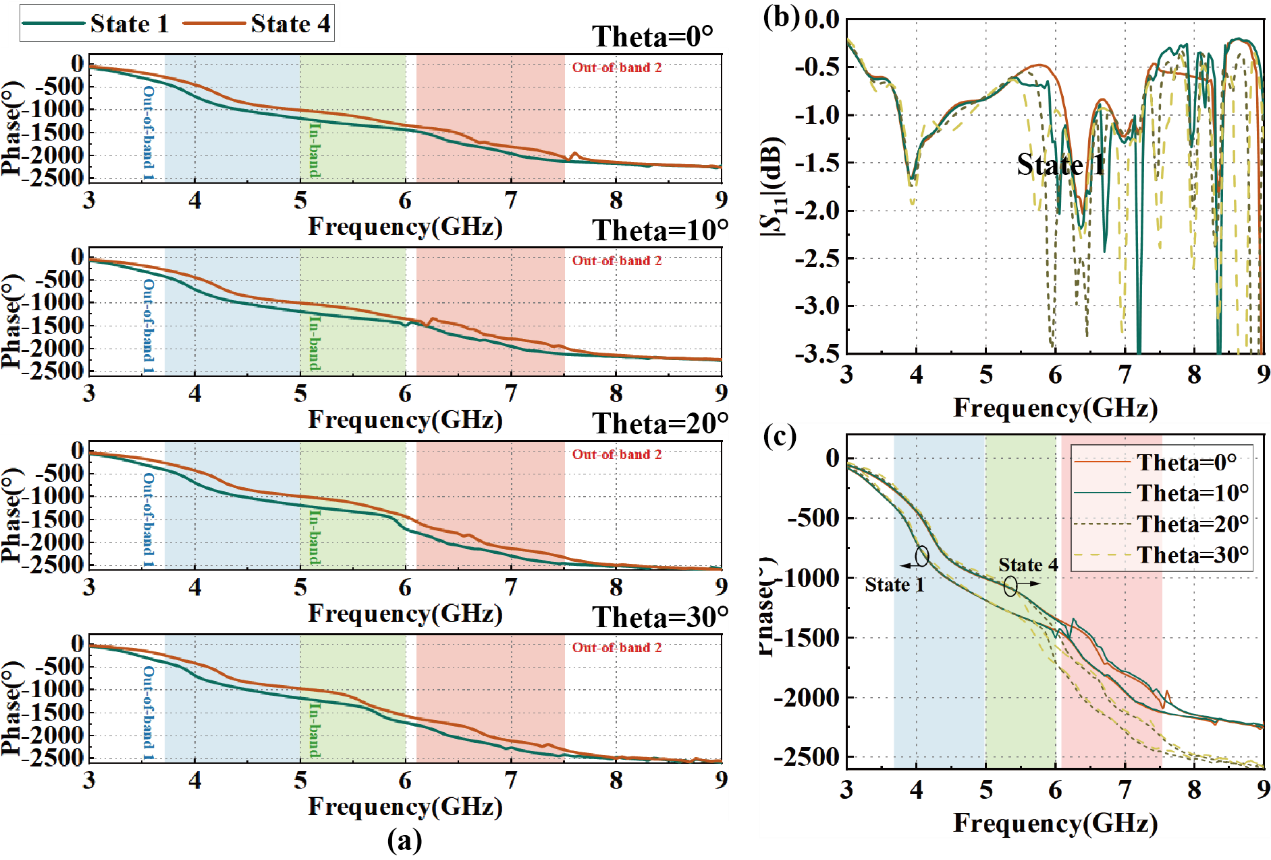


***Fig******ure S12 Reflection coefficient of meta-atom with coating at different incident angles of x-polarization when the coating is 1 mm away from the meta-atom.*** *(a) Reflection phases at different incident angles are displayed separately. (b) Reflection magnitude under state 1. (c) Reflection phases.*

**TABLE S11**

**1-bit phase bandwidth when the coating is 1.5 mm away from meta-atom**

| Theta | Out-of-band1 | In-band | Out-of-band2 | Out-of-band3 |
| --- | --- | --- | --- | --- |
| 0° | 3.6-4.95 | 4.95-6.0 | 6.65-6.85 | 7.6-8.1 |
| 10° | 3.6-4.95 | 4.95-6.0 | 6.65-6.85 | 7.7-8.05 |
| 20° | 3.6-4.95 | 4.95-6.0 | 6.59-6.77 | 7.73-8.05 |
| 30° | 3.6-4.95 | 4.95-5.97 | 6.58-6.75 | 7.77-7.93 |

**TABLE S12**

**1-bit phase bandwidth when the coating is 1 mm away from meta-atom**

| Theta | Out-of-band1 | In-band | Out-of-band2 |
| --- | --- | --- | --- |
| 0° | 3.6-4.95 | 4.95-5.9 | 6.2-7.45 |
| 10° | 3.6-4.95 | 4.95-5.9 | 6.25-7.5 |
| 20° | 3.6-4.95 | 4.95-5.85 | 5.97-7.47 |
| 30° | 3.6-4.95 | 4.95-5.75 | 5.85-7.47 |


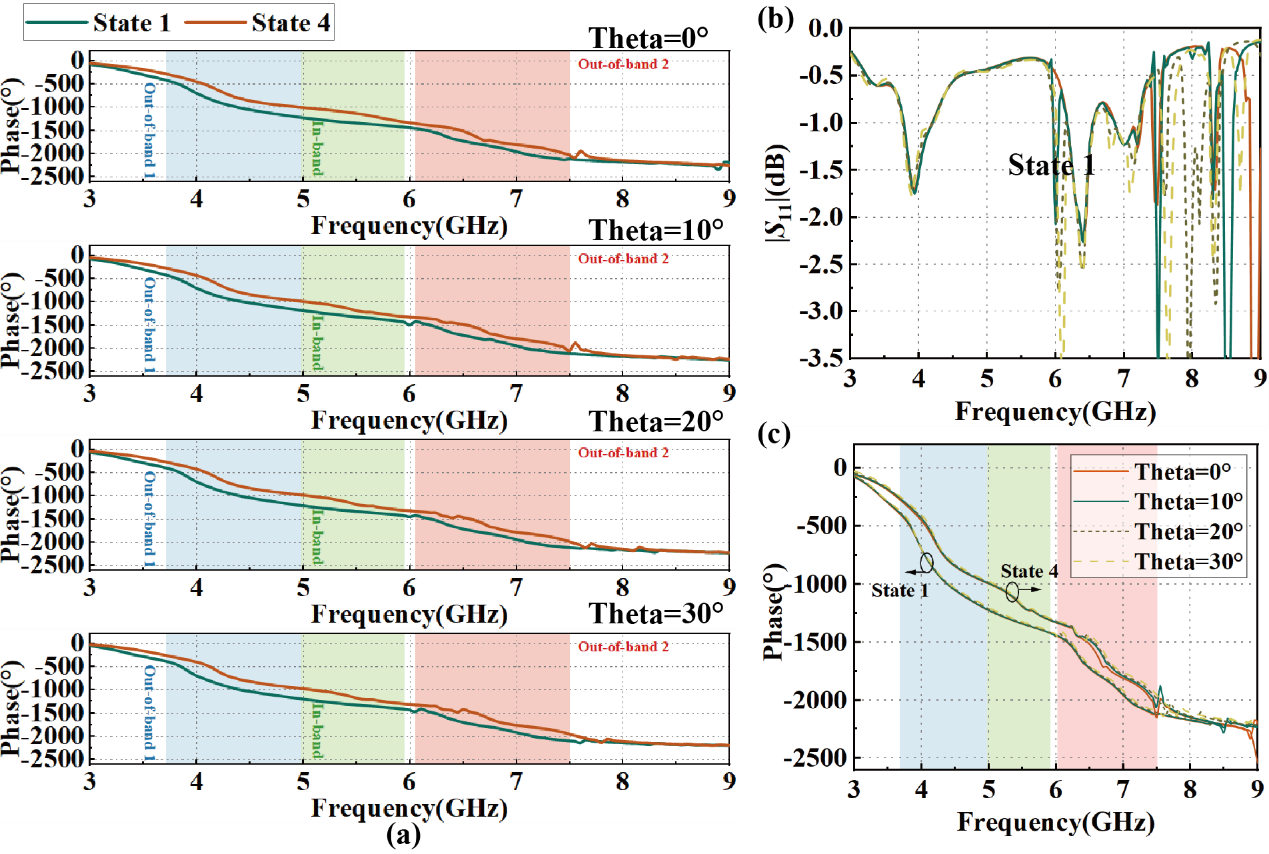


***Figure S13. Reflection coefficient of meta-atom with coating at different incident angles of y-polarization when the coating is 1 mm away from the meta-atom.*** *(a) Reflection phases at different incident angles are displayed separately. (b) Reflection magnitude under state 1. (c) Reflection phases.*

**TABLE S13**

**1-bit phase bandwidth of y-polarization when the coating is 1 mm away from meta-atom**

| Theta | Out-of-band1 | In-band | Out-of-band2 |
| --- | --- | --- | --- |
| 0° | 3.6-4.95 | 4.95-5.9 | 6.1-7.45 |
| 10° | 3.6-4.95 | 4.95-5.9 | 6.1-7.45 |
| 20° | 3.6-4.95 | 4.95-5.9 | 6.1-7.45 |
| 30° | 3.6-4.95 | 4.95-5.9 | 6.1-7.45 |

**Supplementary Note 8. Programmable Metasurfaces Phase Compensation**

For an array beam deflection angle of (*θ*, *φ*), each element requires a continuous compensatory phase:

Where *θ* and *φ* represent the elevation and azimuth angles, respectively. k is the propagation constant, d is the period, and *φ_f_* denotes the phase delay from the feed source to the (m, n) element. The phase delay caused by the feed source can be expressed as:

Where (*x*, *y*, *z*) represents the position of each element. (x0, y0, z0) is the position of the feed source, where *x*_0_ = 0, *y*_0_ = 0, *z*_0_ = F. The focal diameter ratio (FDR) is defined as F/D, where D is the size of the array. After 1-bit quantization, the required compensatory phase for each unit is given by:

The coding pattern for beam pointing at 15°, 30°, and 45° are shown in Figure S14.

(a) (b)

(c)

***Figure S14.*** ***Radiation coding pattern.*** *(a) 15°. (b) 30°. (c) 45°.*

**Supplementary Note 9. Overall Schematics of the Self-Stealth Metasurface**

The layout of the actual processed array is shown in Figure S15. It consists of various components, including the top layer, the middle layer, the RF grounding, and the DC bias network. Different layers are marked with different colors (red, purple, cyan, and blue). The substrate is represented in white. The circular markings around the array represent alignment holes.


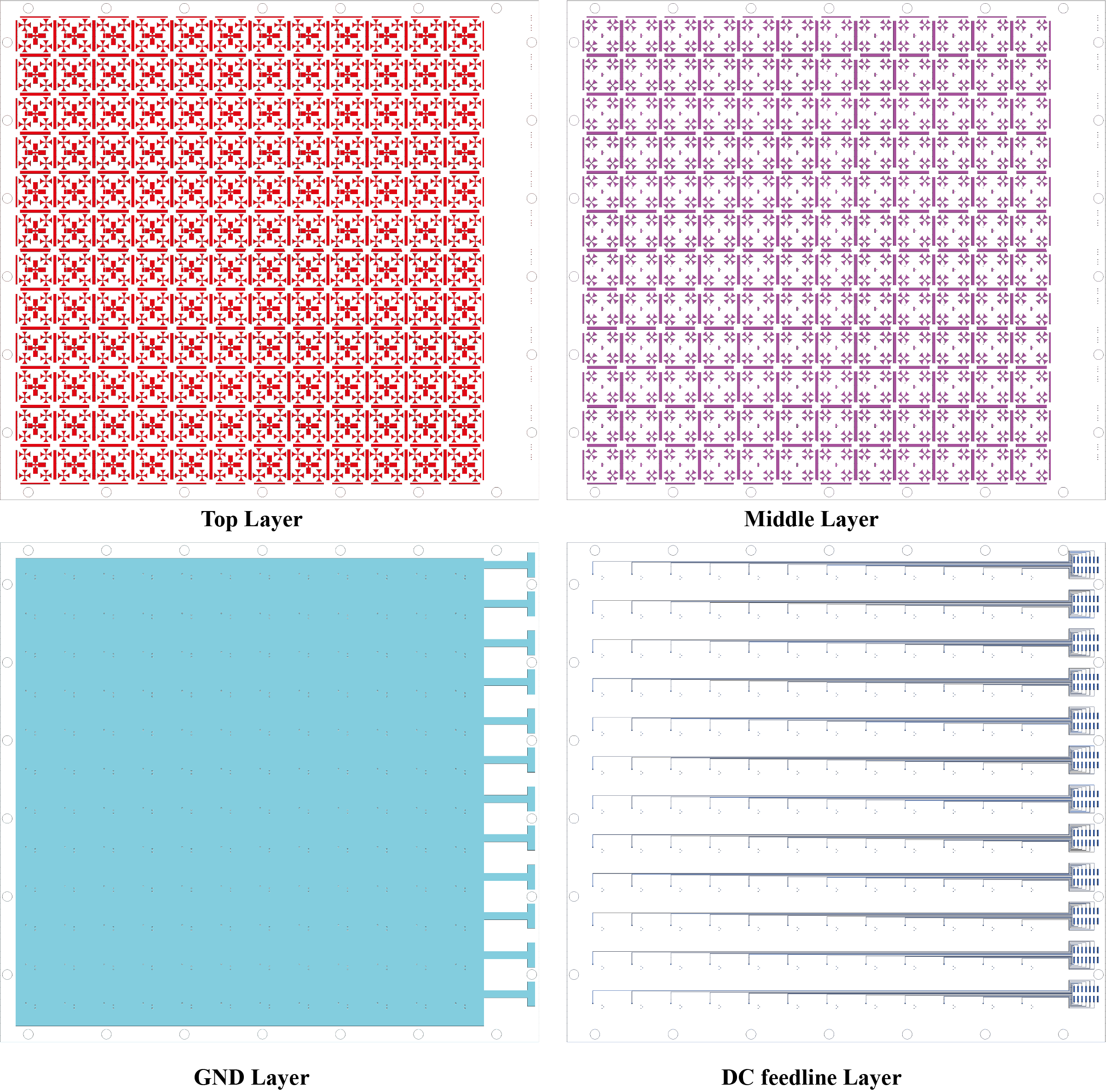


***Figure S15. Schematic illustrations.***

**Supplementary Note 10. Self-stealth Metasurface Prototype**

The top and bottom layers of the processed 12×12 array after soldering are shown in Figure S16. A total of 144 PIN diodes and 144 inductors are soldered on the top of the metasurface. On the bottom layer of the array, twelve sockets are soldered.


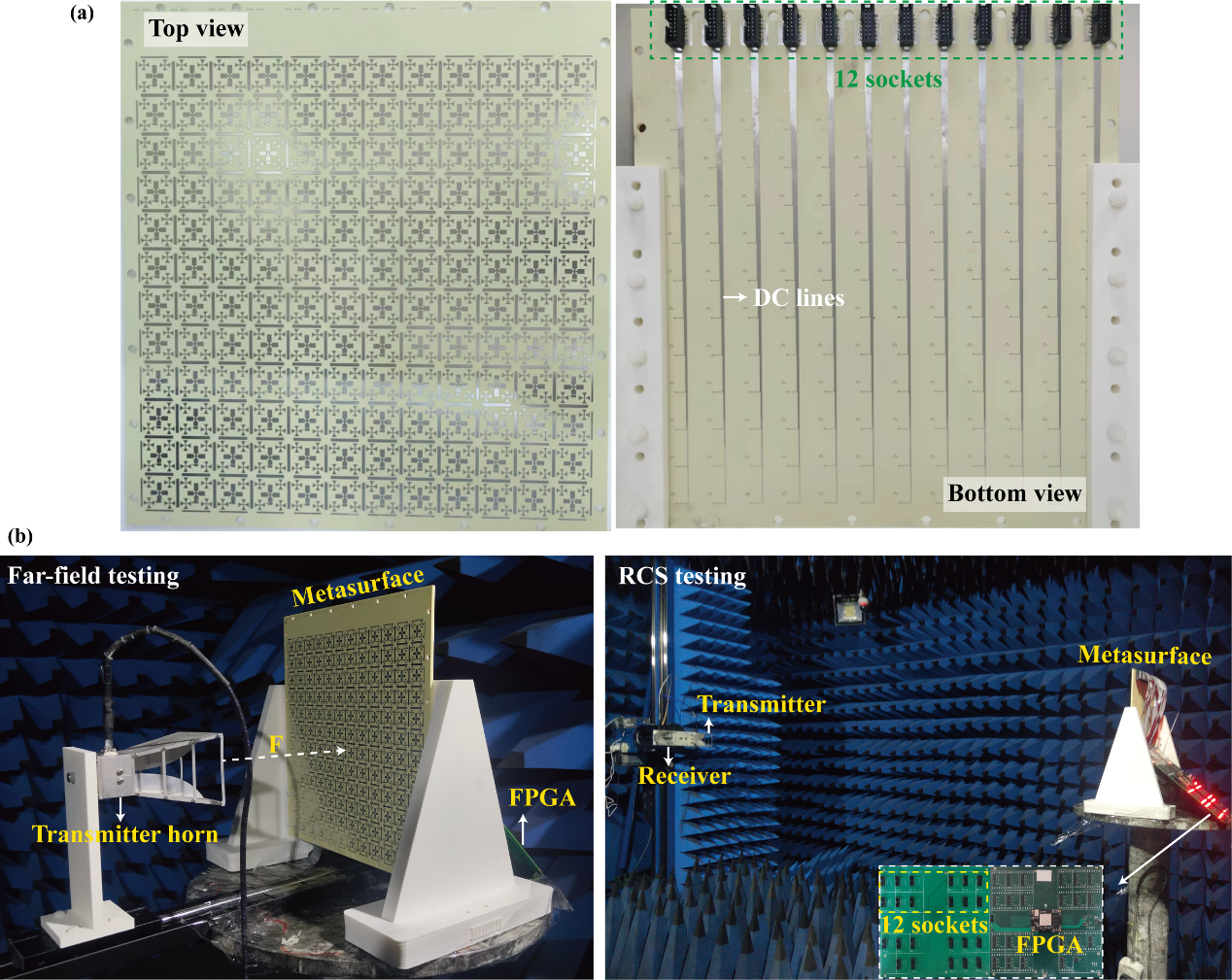


***Figure S16. Top and bottom layers of the processed 12×12 array after soldering.***

**Supplementary Note 11. Measured RCS Reduction Performance for Oblique Incidence**

Figure S17 presents the measured RCSR performance of the metasurface under oblique plane-wave incidence. The results indicate that the 0° coding pattern yields the optimal RCS reduction depth and bandwidth. The RCS reduction performance and bandwidth deteriorate as the angle of incidence increases. As the RCS reduction characteristics at a 30° incidence angle are comparable to those of the metal ground plane, the RCS properties are only provided for incidence angles of 0°, 10°, and 20°.


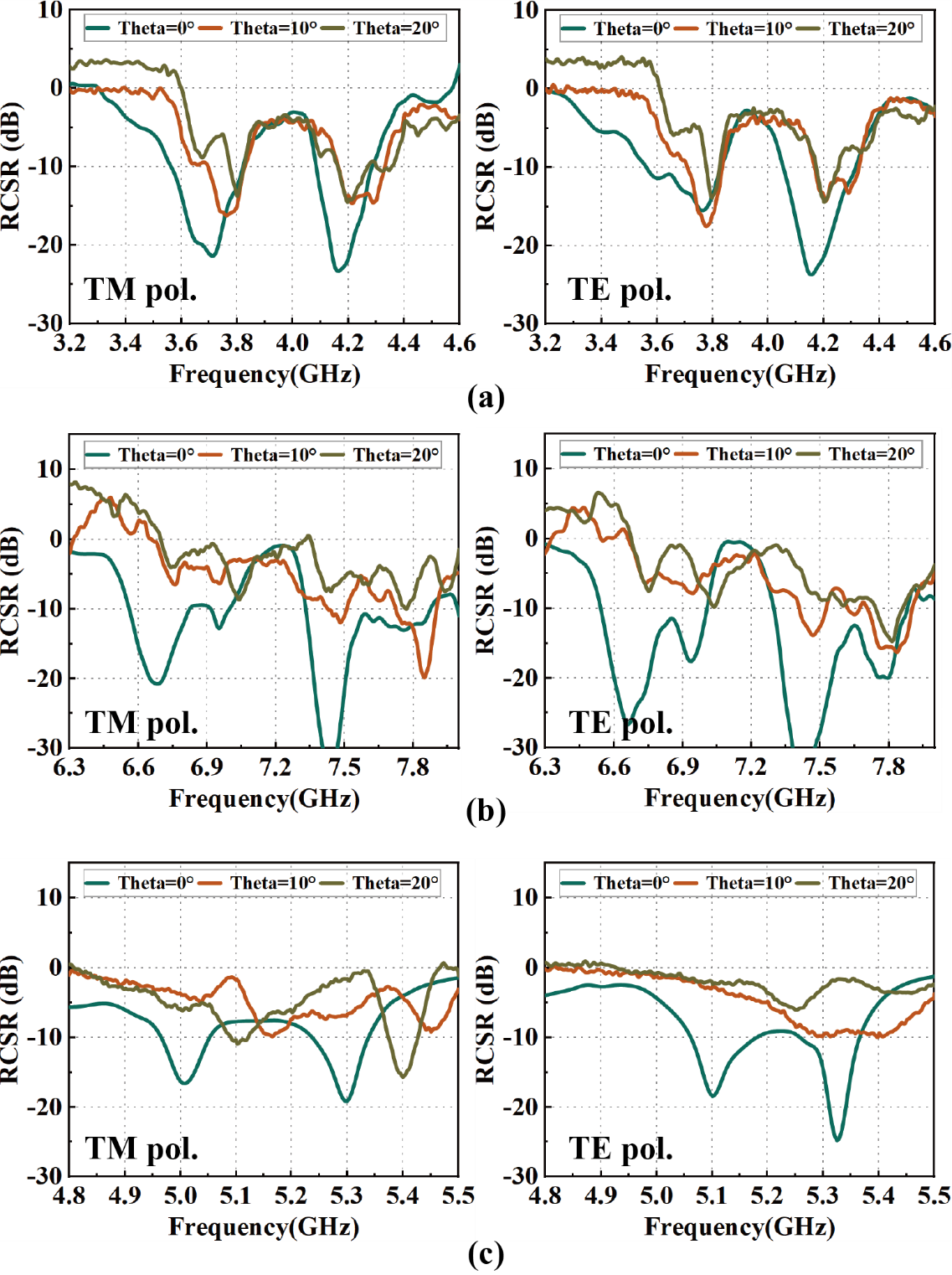


***Figure S17.*** ***RCS reduction.*** *(a) low-frequency out-of-band, (b) high-frequency out-of-band, and (c) in-band.*

**Supplementary Note 12. Simulated In-band Radiation and RCS Reduction Performance of Metasurface with the Coating for Oblique Incidence**

Figure S18 present the beam scanning results in the E-plane and H-plane at 5.2 GHz, respectively. The dielectric coating was positioned 1 mm above the metasurface. It is evident that the programmable metasurface with dielectric coating can achieve good two-dimensional ±60° beam scanning with a scanning step size of 15°. The maximum gain is 18.2 dBi, and the aperture efficiency is 23%. Therefore, the programmable metasurface with coating also exhibits excellent beam scanning characteristics and high efficiency.

The meta-atoms loaded with the dielectric coating were arranged in a checkerboard configuration to simulate their RCS characteristics under oblique incidence. The dielectric coating was positioned 1 mm above the metasurface. The simulated RCSR results are shown in Figure S19. It can be observed that under normal incidence, the -10 dB RCS reduction bandwidth for both x-pol. and y-pol. is approximately 3.6-7.50 GHz (70.71%). at 10° incidence angles, the -8 dB RCS reduction bandwidth for both x-pol. and y-pol. is approximately 3.58-7.50 GHz (70.76%). Furthermore, at 20° and 30° incidence angles, the -6 dB RCS reduction bandwidth for both x-pol. and y-pol. is approximately 3.75-7.50 GHz (70.21%). Therefore, compared to the structure without the coating, the RCS reduction bandwidth under oblique incidence is significantly improved. Moreover, by optimizing the coating, an ultra-wideband RCS reduction characteristic, synthesizing both in-band and out-of-band performance, can be achieved. Thus, adding a wide-angle matching layer directly above the meta-atoms enhances both their bandwidth and wide-angle stability. Additionally, in practical applications, a metasurface requires a protective cover to prevent PIN diode damage and PCB oxidation. Consequently, this protective cover can be designed as a dielectric coating, which not only safeguards the device but also substantially enhances the bandwidth. Essentially, the dielectric protective layer is indispensable for the device. This represents an optimal, most straightforward, and lowest-cost method for bandwidth extension.

**
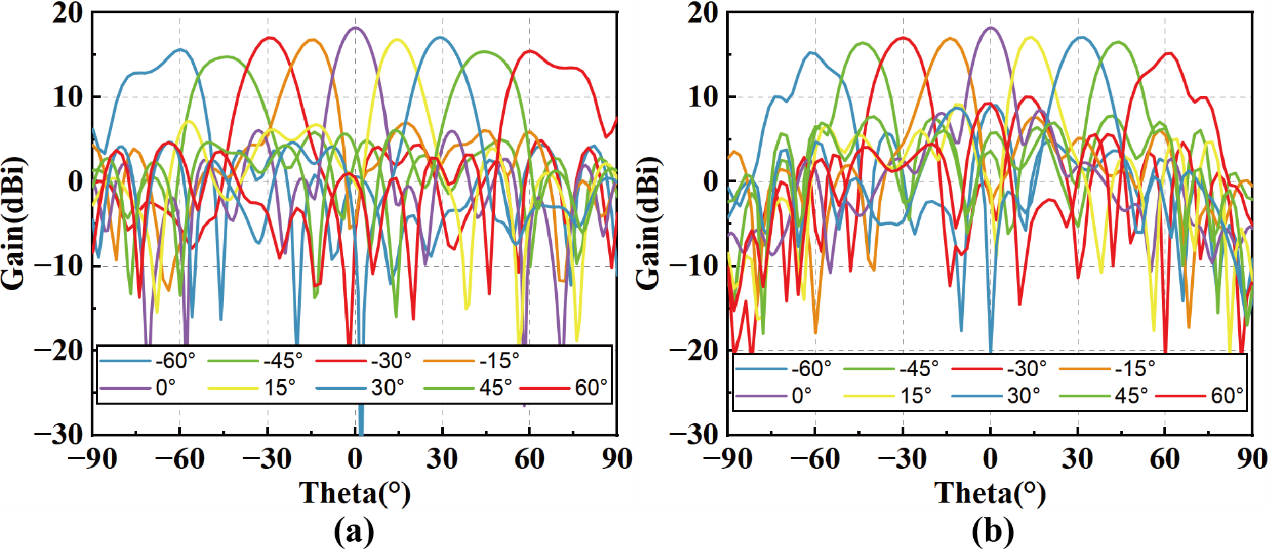
**

***Figure S18.*** ***In-band radiation at 5.2 GHz.*** *(a)E-plane, (b)H-plane.*


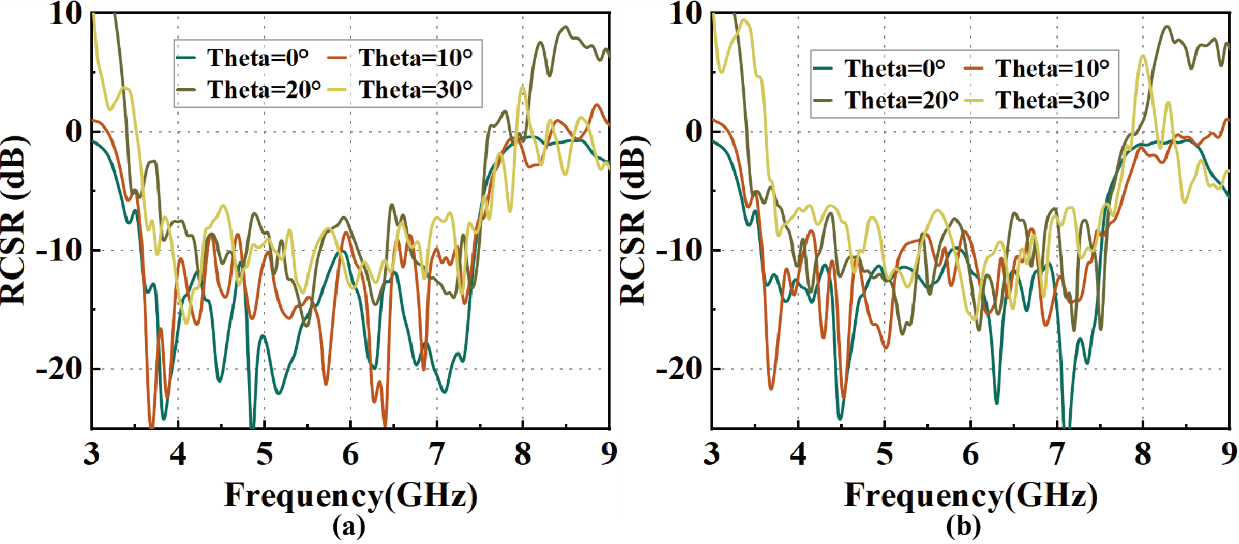


***Figure S19 Simulated RCSR of metasurface with the coating for oblique incidence.*** *(a) x-pol., and (b) y-pol..*

**Supplementary Note 13. Some Issues to be Noted in Practical Applications**

**1. Concerning the Nonlinear Effects and Harmonic Generation in PIN Diodes under High-Power Electromagnetic Wave Illumination**

Theoretically, any semiconductor device, including PIN diodes, can exhibit nonlinear characteristics under extreme conditions, such as high-power incident waves or extremely fast switching transients, thereby generating harmonics. We simulated the nonlinear effects of the meta-atoms under high-power microwave illumination using ADS. First, in HFSS, the RLC boundary condition for the PIN diode within the meta-atom was replaced with a lumped port. The meta-atom was simulated using Floquet ports and periodic boundary conditions. We excited only the x-polarization of the meta-atom. Consequently, only one mode (TM) was considered for the Floquet ports. The S2P file of the meta-atom was obtained from the HFSS simulation. Co-simulation between HFSS and ADS was achieved using this S2P file. Specifically, the S2P file obtained from HFSS was imported into ADS, and the equivalent circuit shown in Figure S1(a) was constructed. The PIN diode was simulated using its SPICE model. We used the SMP1340 diode from Skyworks, whose corresponding SPICE model, downloaded from the official website, is shown in Figure S20(a). An S2P component was added to import the S2P file from the HFSS simulation. Port 1 of the S2P component corresponds to the Floquet port in HFSS, port 2 corresponds to the PIN diode port, and port 3 is grounded. A harmonic balance simulation controller was added to verify the nonlinear efficiency of the meta-atoms under high-power microwave incidence. The harmonic characteristics generated at an incident power of 33 dBm are shown in Figure S20(b) and (c). V1 and V2 represent the voltage values corresponding to different harmonics at the meta-atom's reflection port and the PIN diode port, respectively. It can be observed that, in addition to the fundamental frequency at 5.2 GHz, DC, second harmonic (10.4 GHz), and third harmonic (15.6 GHz) components are present. Higher-order harmonics above the third are negligible due to their very low energy. The voltage levels for the DC, fundamental, second harmonic, and third harmonic components are -4.33 dB, 22.84 dB, 10.56 dB, and 0.021 dB, respectively. Therefore, within the nonlinear response of the PIN diode, the energy of the second harmonic is significantly higher than that of the other harmonics. Although the second harmonic energy under high-power microwave illumination is considerable, typical acquisition radars are generally narrowband systems. Their receivers are sensitive only to signals near their transmitted frequency, *f*_0_. The second harmonic generated by the metasurface lies at 2*f*_0_, a frequency well outside the receiver's bandwidth. Consequently, the acquisition radar itself cannot directly detect the second harmonic generated by its own illumination.

Furthermore, the switching transient of the PIN diode is extremely brief compared to the radar pulse. Therefore, the nonlinear energy generated during the transient process constitutes a very small fraction of the total reflected signal energy. Additionally, when the PIN diode operates in its linear state, we simulated its reflection coefficients for the ON and OFF states in ADS, as shown in Figure S21(a) and (b). In this case, equivalent circuits were used to represent the PIN diode's ON and OFF states. The reflection coefficients obtained from the ADS simulation are shown in Figure S21(c) and (d). It can be observed that the HFSS and ADS simulation results are nearly identical.

**2. Regarding Harmonic Frequencies and Spectral Leakage in Metasurfaces Operating in a Rapid Beam-Switching Mode for Multi-User Communications**

When a metasurface operates in a rapid beam-switching mode, it effectively functions as a temporal modulation, which generates harmonic frequencies. The harmonic frequencies are related to the fundamental frequency and the modulation speed, and are given by:

$$f_{m}=f_{c}+mf_{0}$$

Here, *f*_0_ is the modulation rate (*f*_0_ = 1/T, where T is the period), *f*_c_ is the fundamental frequency, *f*_m_ is the harmonic frequency, and m is the harmonic order. The operating frequency of the programmable metasurface is 5.2 GHz. The modulation rate of the PIN diodes is 10 kHz. Consequently, the frequencies corresponding to the m = -3, -2, -1, 1, 2, and 3 order harmonics are 5.19997 GHz, 5.19998 GHz, 5.19999 GHz, 5.20001 GHz, 5.20002 GHz, and 5.20003 GHz, respectively. Higher-order harmonics possess negligible energy and can be disregarded. This implies that the ±3rd-order sidebands span from 5.19997 GHz to 5.20003 GHz, resulting in a total bandwidth of 60 kHz. Evidently, at a switching rate of 10 kHz, the harmonic frequencies reside extremely close to the fundamental frequency. If the modulation rate is increased to 1 MHz, the corresponding frequencies for the m = -3, -2, -1, 1, 2, and 3 order harmonics become 5.197 GHz, 5.198 GHz, 5.199 GHz, 5.201 GHz, 5.202 GHz, and 5.203 GHz, respectively. This indicates a ±3rd-order sideband range from 5.197 GHz to 5.203 GHz, encompassing a total bandwidth of 6 MHz. Therefore, the sideband bandwidth under temporal modulation is intrinsically linked to the modulation rate. A higher modulation rate yields a broader sideband bandwidth. The metasurface's operational bandwidth within the designated band is 5.05-5.35 GHz. To prevent harmonic sidebands generated by rapid beam switching from falling outside the operational band and compromising stealth performance, the communication bandwidth must be selected according to the modulation rate. For a switching rate of 10 kHz, a communication bandwidth of 5.1-5.3 GHz is selected. The resulting lower and upper harmonic sidebands are 5.09997 GHz and 5.30003 GHz, respectively. Under these conditions, spectral leakage does not occur, thereby preserving the stealth characteristics.


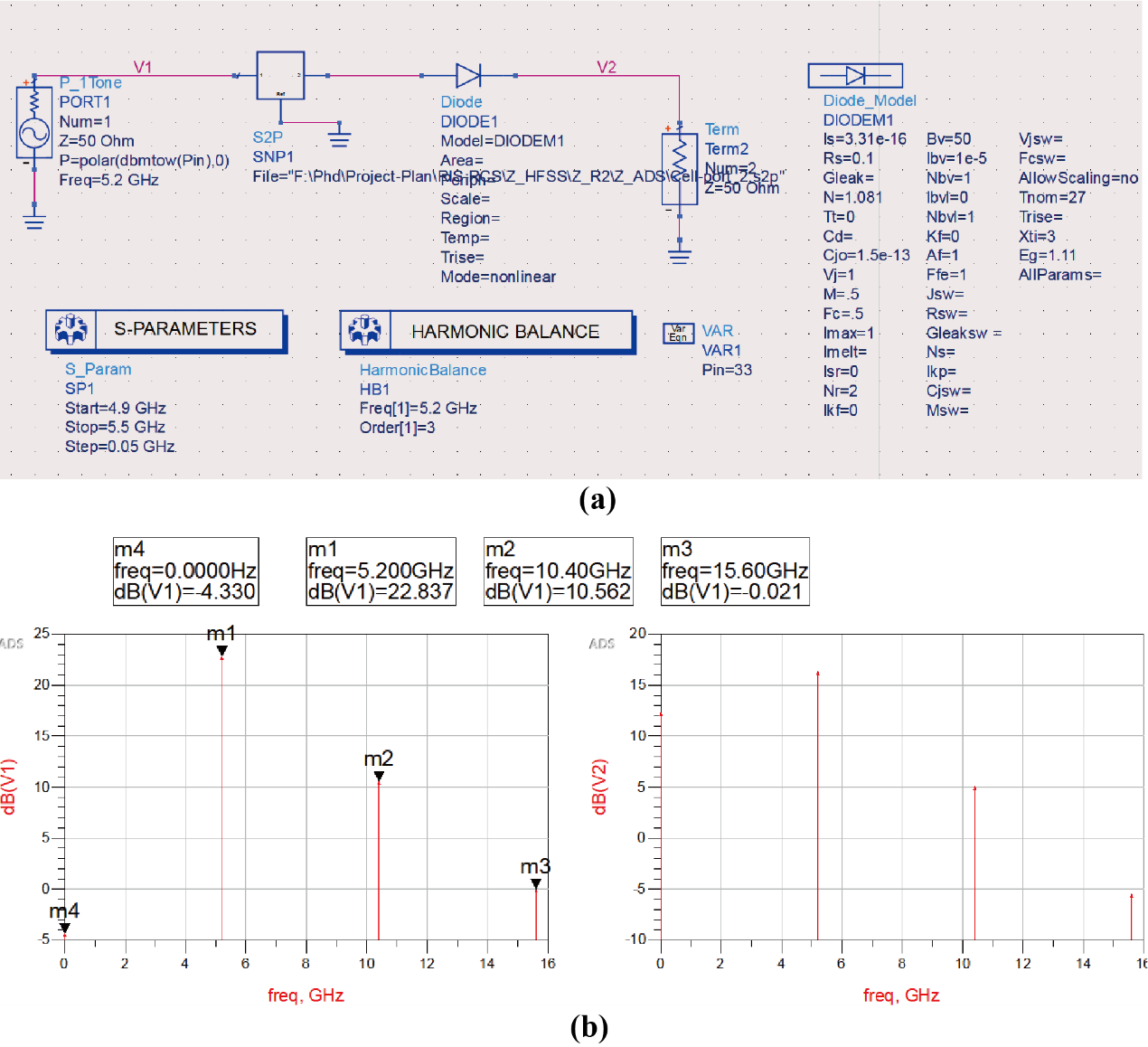


***Figure S20. ADS simulation.*** *(a) Simulation of harmonic characteristics based on equivalent circuit model of meta-atom. (b) Harmonic characteristics.*


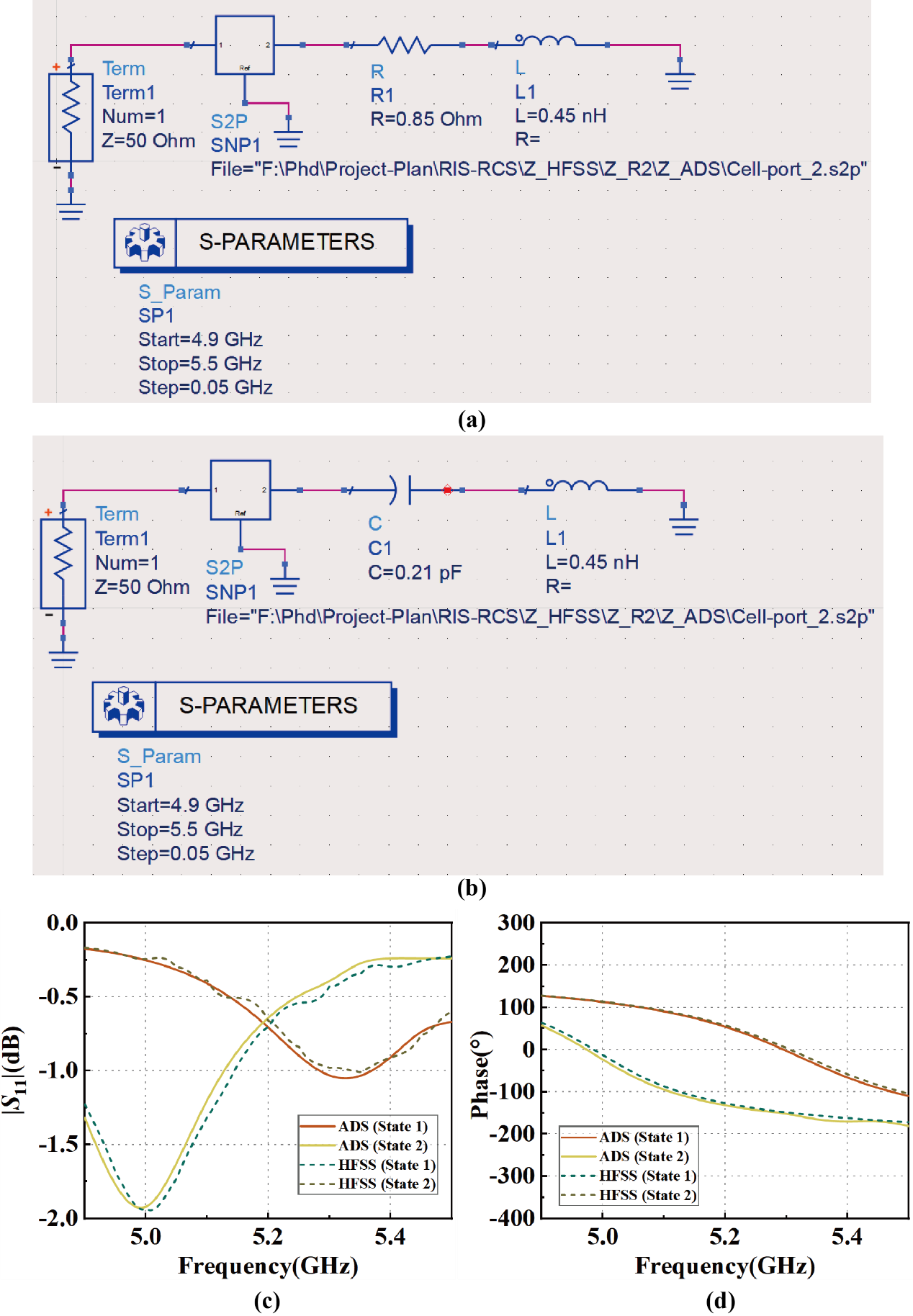


***Figure S21. ADS simulation.*** *Equivalent circuit model with (a) ON, and (b) OFF states. Reflection coefficient of (c) magnitude, and (d) phase.*
